# Pathogen-phage geomapping to overcome resistance

**DOI:** 10.7554/eLife.109259

**Published:** 2026-08-06

**Authors:** Camilla Do, Keiko Christine Salazar, James D Chang, Justin R Clark, Austen Lee Terwilliger, Paul Ruchhoeft, Paul Nicholls, Anthony W Maresso

**Affiliations:** 1 Department of Molecular Virology and Microbiology, Baylor College of Medicine Houston United States; 2 TAILΦR LABS, Baylor College of Medicine Houston United States; 3 Department of Electrical & Computer Engineering, University of Houston Houston United States; 4 Section of Infection Diseases, Department of Medicine, Baylor College of Medicine Houston United States; CorpoGen Colombia; University of the Witwatersrand South Africa

**Keywords:** bacteriophages, Pseudomonas phages, Escherichia phages, Klebsiella phages, enterococcus phages, Viruses

## Abstract

The rise of antibiotic resistance has renewed interest in bacteriophages as therapeutic alternatives. However, coevolution of phage and bacteria will naturally give rise to phage-resistant pathogens, complicating phage therapy efforts. A critical bottleneck in the production of phage therapeutics is the discovery of virulent phages against resistant pathogens. Conventional methods for discovery are time-consuming, biased, and laborious, limiting the potential for identifying suitable phage candidates. To overcome these limitations, we combined small-volume environmental sampling with 16 S rRNA sequencing to identify reservoirs where bacterial hosts co-exist with their phage predators. This strategy, which we term geographical phage mapping (geΦmapping), pinpoints ecological ‘hotspots’ for targeted phage hunting. We further developed a portable phage hunting device (ΦHD) that generates highly enriched phage concentrates directly from these reservoirs. By integrating geΦmapping with high-throughput enrichment, we constructed the RΦ library, a diverse collection of novel phages. We captured and isolated 36 new phages targeting extremely resistant organisms across various ESKAPE pathogens when conventional phage hunting and experimental evolution approaches failed.

## Introduction

**Figure 1.**
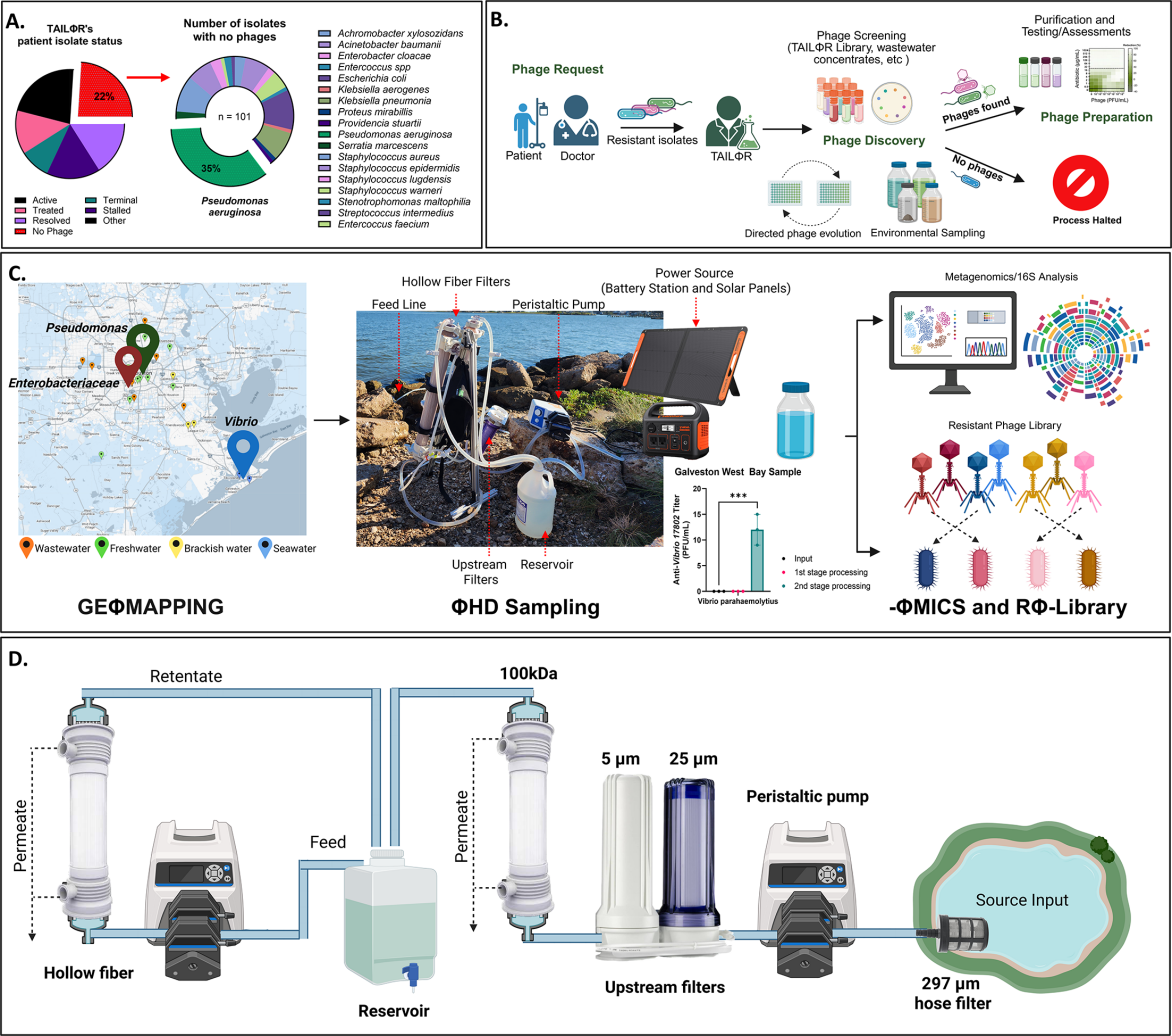
Overview and schematic of ΦHD. (**A**) Pie charts of patient isolate status and percentage of bacterial strain with no phages for in TAILΦR’s library. Unfortunately, 24% of isolates that TAILΦR receives have no phages; at 35%, most of the isolates are *Pseudomonas aeruginosa*. (**B**) Diagram from phage request to phage discovery and limitation. Clinicians and their patients can seek phage therapy for antibiotic-resistant infections when there are no other approved treatments available. After approval, clinicians send patient isolates to TAILΦR. TAILΦR’s phage library and wastewater concentrates are screened against the patient isolate. If phages are discovered, they are moved onto the next stage for preparation where phages are ultimately purified and tested for safety and efficacy before being handed to the clinician. When there are no phages for that isolate in the library, TAILΦR searches for phages through environmental sampling. Alternatively, they can train related phages to infect the isolate through directed phage evolution. (**C**) Diagram of strategies employed in our study. Geographical phage (Φ) mapping (geΦmapping) allowed us to pinpoint target sites rich in our pathogen of interest from our environment (*left*). Once a location is chosen, we used our capture device, the phage hunting device (ΦHD), to filter, concentrate, and enrich our sample with phage and bacteria (*middle*). Bar graph shown here was derived from Figure 2F as an example for phage concentration. With the samples, we further analyzed them with phage metagenomics (ΦMICS) and screened, isolated, and characterized phage present in the samples (*right*). This led to the curation of the resistant phage (Φ) library (RΦ-Library). (**D**) Schematic of ΦHD. The components on the right of the reservoir draw, filter, and concentrate water through ΦHD. On the left of the reservoir, the remaining component concentrates any material in the reservoir. Created with BioRender.com.

The rise of antibiotic resistance has brought global healthcare to the precipice of a post-antibiotic era, driving interest in bacteriophage (phage) therapy as an alternative strategy to combat resistant bacterial infections (Naghavi et al., 2024; Pirnay et al., 2024; Green et al., 2023). Unlike antibiotics, phages evolve with their host; both locked in a relentless evolutionary arms race. Bacteria and phage continuously expand their repertoires of resistance and counter-resistance strategies. Among many examples, phages counter bacterial defenses by exposing receptors with enzymes, evading restriction enzymes with DNA base modification, and evolving anti-CRISPR proteins (Hutinet et al., 2019; Katz et al., 2024). In response, bacteria resist phage infection by blocking adsorption and nucleic acid injection and using restriction enzymes and CRISPR-Cas9 to target phage nucleic acids (Garneau et al., 2010; He et al., 2024). This dynamic interplay of phage resistance poses a barrier to effective phage therapy (Oechslin, 2018). TAILΦR is a phage center dedicated to providing lytic phages to physicians to treat patients battling multidrug-resistant infections (Terwilliger et al., 2020). Across 263 distinct cases encompassing 462 bacterial isolates, 22% of isolates given to TAILOR remain untreated despite an extensive library of over 325 phages targeting 15 bacterial species (Figure 1A). Information regarding TAILΦR’s phage and bacterial library can be found in Supplementary files 1–3. When no phages in the library can target a pathogen, TAILΦR turns to directed evolution (to expand the host range of a given phage) or environmental sampling (to find natural phages). Such approaches often fail to yield a suitable phage, and so discovering new, virulent phages that can effectively target these pathogens becomes a critical and rate-limiting step (Figure 1B). As phage therapy spreads, resistant isolates will inevitably emerge under increased selection pressure, analogous to antibiotic resistance after the introduction of penicillin (Rammelkamp and Maxon, 1942). This puts the onus onto phage discovery where improvements are critically needed (Olsen et al., 2020b).

Phage engineering is one method of constructing phages for improved therapeutic potential. Examples include generating lytic derivatives of lysogenic phages and altering phage tail fibers to adjust their host range (Dedrick et al., 2019; Yehl et al., 2019). In contrast, phage-host competition has naturally addressed phage resistance over billions of years of evolution. The challenge lies in isolating these phages from nature. Conventional methods for phage discovery can be time-consuming or introduce bias (Olsen et al., 2020b). Phage hunters are limited to sampling small volumes of water, a constraint that typically necessitates the use of enrichment techniques to increase the likelihood of phage detection (Sada and Tessema, 2024; Van Twest and Kropinski, 2009; Aghaee et al., 2021; Kenney and Gómez-Duarte, 2024). Enrichments can favor fast-growing or highly virulent phages, potentially overlooking those that may be therapeutically relevant but less abundant or slower to propagate (Muniesa et al., 2005). To sample larger volumes, dead-end filtration (limited by low filtration speed) and iron chloride flocculation (limited by phage inactivation) are typically used (Supplementary file 4). Roux et al., 2016; Gios et al., 2024; Xiong et al., 2023; Chen et al., 2023; Labbé et al., 2020; Malki et al., 2020; Angly et al., 2006; Schoenfeld et al., 2008; Gu Liu et al., 2024; Heffron et al., 2019; Langenfeld et al., 2021 The speed and phage bias limitations are acceptable for metagenomic approaches but unhelpful for phage therapy, leaving a critical gap for patients that urgently need phages for phage-resistant bacterial infections.

Viral metagenomics involves identification of largely uncultivated viral genomes from the environment. Unfortunately, analysis is hindered by the vast diversity of viruses, lack of consensus sequences, and limited reference genomes available (Roux et al., 2019). Some 70% of all viral genomes are unclassified, often referred to as ‘viral dark matter’ (Roux et al., 2015). Studying unknown viral genomes can advance viral ecology and may contribute useful gene products for research and therapeutics (Huss et al., 2025). Viral metagenomics are frequently limited by low biomass, sampling biases, and limitations in sampling remote sites (Thurber et al., 2009; Gowers et al., 2019).

In this study, we sought to improve current methods for phage discovery to tackle phage-resistant pathogens and enhance gene discovery. We used a bioprospecting approach, collecting samples in East Texas, USA, and applied 16 S rRNA analysis to identify pathogenic reservoirs and hence their phages. We dubbed this process, ‘Geographical Phage (Φ) Mapping,’ or ‘GeΦmapping.’ After identifying pathogen-rich sources, we deployed a high-throughput phage hunting device, ΦHD, for sample processing (Figure 1C and D). We used this approach of combining molecular bioprospecting and high-volume sampling to address problematic ‘extremely phage-resistant organisms’ (XΦROs) in clinical cases.

## Results

### GEΦMAPPING of water sources from Houston and Galveston, Texas

**Figure 2.**
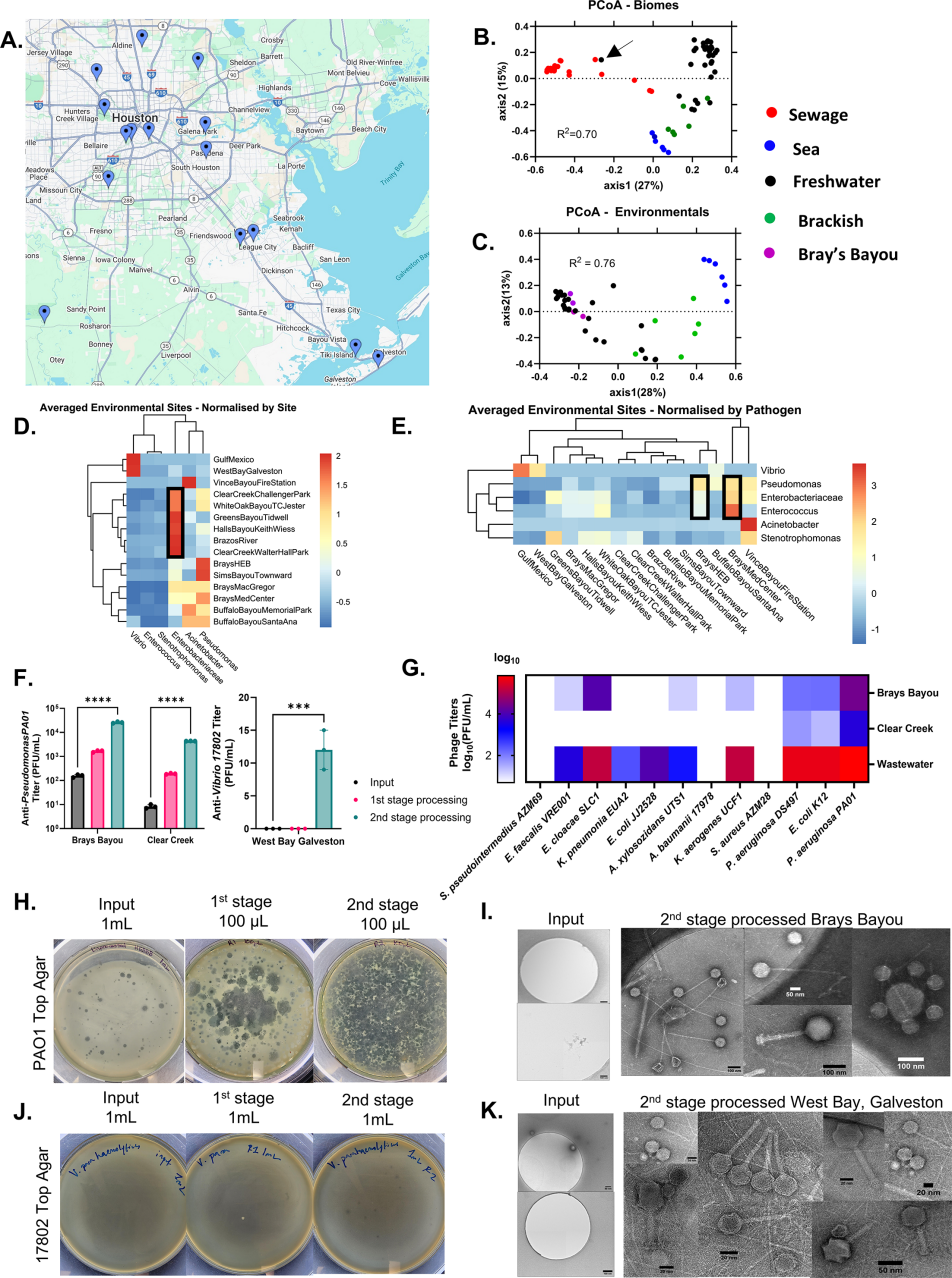
GEΦMAPPING highlights Bray’s Bayou as a *Pseudomonas*-rich target site, and performance of ΦHD sampling of several environmental sites. (**A**) Geographical map of freshwater samples obtained in Houston, TX, USA. (**B**) Principal Coordinate Analysis (PCoA) analysis showing β-diversity differences between sewage, sea, and freshwater sources. The black arrow indicates the Vince Bayou sample. (**C**) PCoA analysis (Bray–Curtis dissimilarity) of environmental water bodies with Bray’s Bayou target sites highlighted (purple). (**D**) Heat map with z-score normalized by site shows distinct patterns of microbial prevalence at various sites. (**E**) Heat map, z-score normalized by pathogen, highlights the potential of Bray’s Bayou as a target site for *Pseudomonas* and enteric phages. (**F**) Quantification of anti-Pseudomonas (left) and anti-Vibrio (right) plaques (PFU/mL) from different stages of processing (input, first stage, and second stage processing). (**G**) Heatmap showing anti-pathogen phages (log_10_[PFU/mL]) from freshwater sites. We display the mean and SEM of three technical repeat measurements. (**H**) Plaque assay on *P. aeruginosa* PAO1 of unprocessed, first stage concentrate, and second stage concentrate of a freshwater sample. (**I**) TEM of unprocessed water from freshwater. (**J**) Plaque assay on *Vibrio parahaemolyticus* 17802 of unprocessed, first stage concentrate, and second stage concentrate seawater sample. (**K**) TEM of unprocessed water from seawater, respectively. Representative images shown (**H–K**). Statistical analysis. (**F**, left panel) Phage titers were compared using a two-way ANOVA with Dunnett’s multiple comparisons test (*α*=0.05; n=3 technical replicates per condition), revealing significant effects of sampling site (F(1,12) = 419.7, p<0.0001), processing stage (F(2,12) = 670.2, p<0.0001), and their interaction (F(2,12) = 338.3, p<0.0001), indicating that the effect of processing stage differed between sites. Second-stage processing significantly increased phage yield relative to unprocessed input at both Bray’s Bayou (mean difference = 25,744 PFU/mL, p<0.0001) and Clear Creek (mean difference = 4,325 PFU/mL, p<0.0001); first-stage processing did not reach significance at either site (p=0.075 and p=0.945, respectively). (F, right panel) Phage titers across processing stages were compared using a one-way ANOVA with Dunnett’s multiple comparisons test (*α*=0.05; n=3 technical replicates per condition), revealing a significant effect of processing stage (F(2,6) = 48.00, p=0.0002). Second-stage processing yielded significantly higher titers relative to unprocessed input (p=0.0003), while first-stage processing did not differ significantly from input (p>0.9999). We display the mean and SD of three replicates for both graphs.

Phages are found where their hosts thrive (Clokie et al., 2011). In marine ecosystems, for example, phage-to-bacterium ratios often reach 10:1, reflecting close associations between bacterial density and phage populations (Chibani-Chennoufi et al., 2004). We identified sites of high pathogen abundance to determine where to hunt for phages. We sampled 15 waterways around Houston and Galveston, Texas, USA thrice and performed 16 S rRNA analysis to examine the bacterial constituents (Figure 2A). We performed rarefaction analysis to estimate species richness relative to sampling effort. The logarithmic shape of the rarefaction curve indicates that most of the abundant taxa present have been detected in each sample, although sample saturation was not fully achieved (Figure 2—figure supplement 1A, top). To further quantify species richness, we compared α-diversity indices (inverse Simpson, Shannon, and Chao) and noticed that White Oak Bayou was the richest (Figure 2—figure supplement 1B). To compare the membership of each site’s microbiome, we analyzed β-diversity using principal coordinate analysis (PCoA) based on Bray–Curtis dissimilarity (Figure 2B and C). Samples of seawater, sewage, and freshwater clustered distinctively, whereas samples from brackish water mingled between freshwater and seawater sites (Figure 2B and C). We performed an analysis of molecular variance (AMOVA) to compare samples derived from brackish, sea, fresh, and sewage. AMOVA revealed significant structuring across all habitat comparisons, with among-group variance exceeding within-group variance in every case. Differentiation was strongest between fresh and sewage samples (Fₛ=28.71, p<0.001), followed by sea-sewage (Fₛ=20.21, p<0.001) and brackish-sewage (Fₛ=15.92, p<0.001), indicating pronounced divergence associated with wastewater. Comparisons involving brackish with seawater (Fₛ=4.65–13.16, p≤0.006) and brackish-fresh (Fₛ=5.96–13.16, p≤0.001) were also significant (Fₛ=4.65–13.16, p≤0.006). Interestingly, a freshwater sample from Vince Bayou, where *Acinetobacter* was prominent, clustered with sewage samples suggesting its microbial profile resembled that of sewage. Of note, a contaminated industrial facility, formerly an oil processor and wastewater treatment plant, is situated upstream of this location (Environmental Protection Agency, 2024). After heavy storms, toxic wastes have reportedly contaminated the area with reports of hundreds of dead fish (Kelly, 2024). During sampling, toxic chemical contamination may have shifted the microbiome to resemble sewage-like communities, causing resilient genera like *Acinetobacter* to dominate.

**Figure 2—figure supplement 1.**
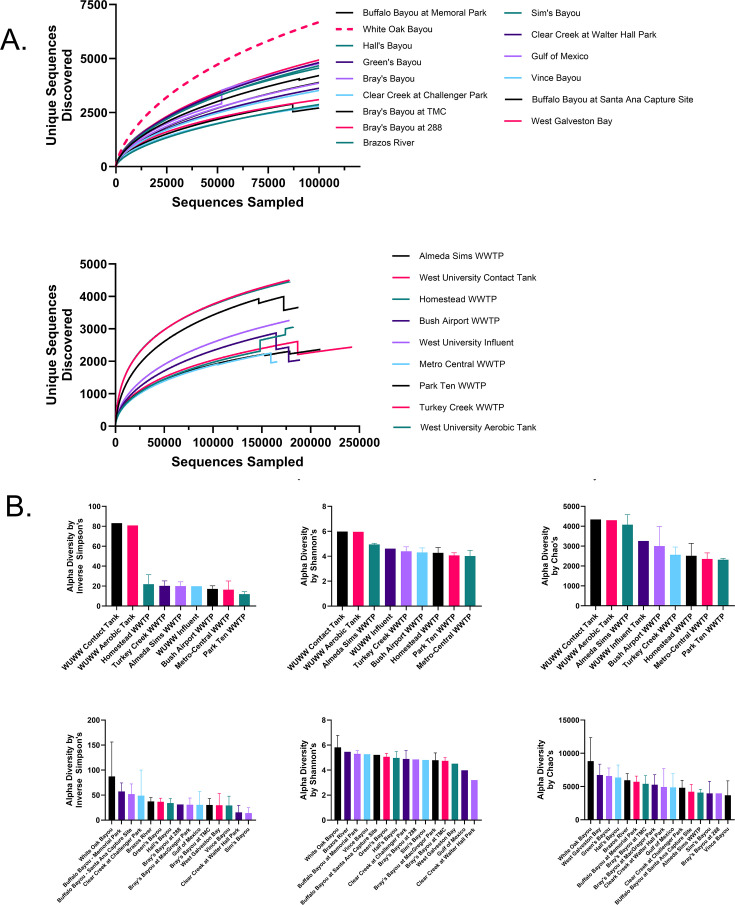
16 S analysis shows adequate sampling (rarefaction curves) and high diversity (α-diversity indices) for sampling collection. (**A**) Rarefaction curves for each site show no sites were under-sampled in environmental sites (*top panel*) or sewage sites (*bottom panel*). Curves represent the mean of triplicate biological samples for all sites except for West University WWTP. Rarefaction was performed in MOTHUR with the default 1000 randomizations. (**B**) α-diversity indices for sewage sites only (*top panel*) and environmental sites only (*bottom panel*). Left panels show the Inverse Simpson’s Index, middle panels show the Shannon index, and right panels show the Chao’s index. Averages represent biological triplicates, except for West University measurements where only single replicates were available, and error bars show SEM. α-diversity calculations were made using MOTHUR where we standardized the calculation to 50,000 random sequences for each group.

**Figure 2—figure supplement 2.**
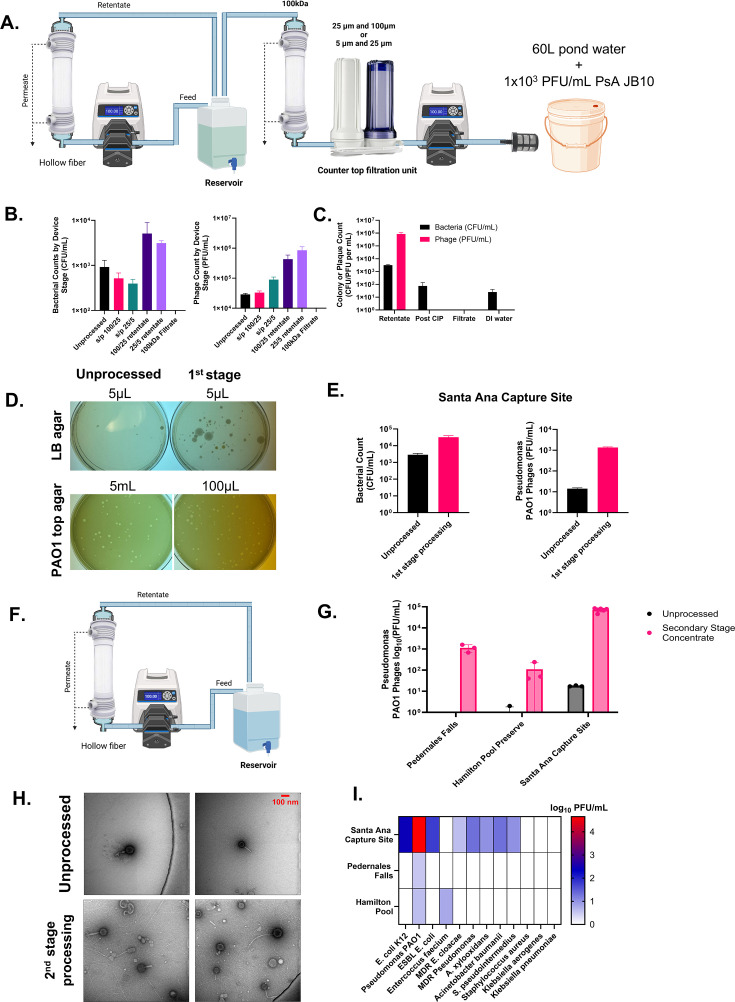
Spike-in and early on-field ΦHD sampling experiments. (**A**) Schematic of ΦHD spike-in study where 60 L of pond water was spiked with 1x10^3^ PFU/mL of *Pseudomonas* phage JB10. We used two set-ups with changes in the upstream filters: 100/25 µm or 25/5 µm set-up. (**B**) Bacterial counts (*left*) and phage titers (*right*) were measured for each upstream filtration (status post s/p 100/25 or s/p 25/5) and after concentration with ΦHD (retentates). Indicator strain used was *P. aeruginosa PAO1*. (**C**) Validation of cleaning in place (CIP) protocol on PAO1 after spike-in study. Mean and SEM from two to four technical replicates are shown. With ΦHD, we performed our field test at Santa Ana Capture Site (SACS). (**D**) Representative plates of unprocessed and first stage concentrated samples from the SACS, plated on LB agar or Pseudomonas PAO1 lawns. (**E**) Quantification of anti-Pseudomonas plaques and bacterial colonies from SACs from two separate runs. We incorporated an in-lab, benchtop concentration step to our ΦHD retentates. (**F**) Schematic of second concentration phase of the ΦHD system in-lab. Using ΦHD, we sampled freshwater from Pedernales Falls (PF), Hamilton Pool (HP), and SACS. (**G**) Quantification of anti-Pseudomonas phages from PF, HP, and SACS before processing and after second stage processing. (**H**) TEM imaging shows sparse virus-like particles (VLPs) in unprocessed SACS samples compared to concentrated VLPs in SACS retentate samples. Representative images shown. (**I**) Heat-map showing anti-pathogen phages from each sampled site. We display the mean and SEM of three technical repeat measurements.

To focus on pathogens, we extracted the counts of pathogen-related taxa and generated heatmaps normalized by site (Figure 2D) and by taxa (Figure 2E). By highlighting selected pathogenic taxa, we can compare patterns across the environments relative to the other taxa; this does not imply absolute abundance or general enrichment of these taxa in the environment. Genus-level classification includes both pathogen and non-pathogenic species. Normalizing by site, we determined the microbial composition of each location. We detected *Vibrio* in Gulf of Mexico and West Bay; both expected in marine environments (Figure 2D). We noted *Enterobacteriaceae* was most common in waterways draining rural and suburban areas, while *Pseudomonas* was more common in larger urban waterways (Brays and Buffalo Bayous; Figure 2D, black rectangles). Again, Vince Bayou was an outlier, highly enriched with *Acinetobacter*.

Normalizing by taxa allowed us to find sites of interest for ΦHD sampling (Figure 2E, black rectangles). The data showed predominance of *Vibrio* at marine sites, while *Pseudomonas*, *Enterobacteriaceae,* and *Enterococcus* were best represented at Brays Bayou. Vince Bayou remained enriched with *Acinetobacter*. Of the 22% of TAILΦR isolates without a phage, 35% of these were *Pseudomonas* (Figure 1A, Supplementary file 3). This made Brays Bayou, rich in *Pseudomonas,* an ideal target for high volume sampling.

### Environmental sampling freshwater and seawater with ΦHD

Although phages are abundant in nature, harvesting phages against pathogens can be difficult (Sada and Tessema, 2024; Kenney and Gómez-Duarte, 2024). Typically, phage hunters are restricted to small volumes and sometimes rely on enrichment techniques to compensate (Sada and Tessema, 2024; Kenney and Gómez-Duarte, 2024). These techniques vary by sample and host but take days with no guaranteed success. After selecting our sampling location, the next challenge was obtaining a highly concentrated sample through high-volume sampling to ensure sufficient phage abundance for effective downstream screening.

We approached this using ΦHD, which was inspired by the various research groups that used tangential flow filtration (TFF) for viral metagenomics (Angly et al., 2006; Schoenfeld et al., 2008; Fulton et al., 2009). We designed ΦHD to have a robust filtration train combined with a high-flux concentration component, allowing for low shear fluid path and high portability (Figure 1D). We used a 297 μm hose filter to screen out larger debris, followed by sequential 25 and 5 µm filter cartridges to remove larger microorganisms such as plankton and protists. We then used two hollow fiber TFF units, operating in parallel for concentration with flow rates in the low shear region. The first unit performs ultrafiltration and fills the reservoir with retentate, whereas the second takes from that reservoir and continuously concentrates the retentate in a closed loop. These are powered by a dual-headed peristaltic pump equipped with a lithium-ion battery and solar panels to allow prolonged operation in the field. During spike-in studies, we spiked pond water (60 L) with phage (1×10^3^ PFU/mL of ΦJB10; Figure 2—figure supplement 2A). We found minimal losses of phage and bacteria during passage through the filtration train and obtained an average 20-fold increase in phage yield (Figure 2—figure supplement 2B). After the spike-in study, we evaluated our cleaning-in-place protocol (Figure 2—figure supplement 2C, Post CIP) for the TFF units and found it effectively removed phages and bacteria, confirming ΦHD is reusable.

In the field, we routinely achieved flow rates of 200 L/hr and gathered samples ranging from 400 to 1200 L (Supplementary file 5). Initial testing on-site proved the filtration/concentration train was robust enough to manage the highly polluted waterways of Houston (Figure 2—figure supplement 2D–E, Santa Ana Capture Site [SACS]). Although not all *Pseudomonas* phages will be detected, we selected *Pseudomonas aeruginosa* PA01 as the indicator strain because of its broad susceptibility profile and its role as a permissive host. After first stage processing with ΦHD, phage yield increased by 95-fold in comparison to unprocessed sample (Figure 2—figure supplement 2E, right panel). We also sampled the more pristine waters of Pedernales Falls (PF) and Hamilton Pool Preserve (HP), and incorporated a secondary in-lab concentration stage to further increase retentate concentrations (Figure 2—figure supplement 2F). Phage yields from inputs from PF and HP were near zero but increased to 10^2-3^ PFU/mL with secondary processing. Similar treatment of SACS retentates yielded an average 3501-fold increase in phage yield (Figure 2—figure supplement 2G). This shows ΦHD, with second stage concentration, greatly increased the detection of viable phages from a given site (Figure 2—figure supplement 2G and H). Screening with other bacteria showed more phages against other pathogens from polluted SACS water compared to pristine sites (Figure 2—figure supplement 2I).

To test the combination of geΦmapping and ΦHD, we selected one site that was highlighted by our map as *Pseudomonas-*, *Enterobacteriaceae-,* and *Enterococcus*-rich (Brays Bayou, Figure 2E) and one low-richness site (Clear Creek). We processed 400 L/site and tracked phage yields by endogenous anti-*Pseudomonas* (PAO1) phages. ΦHD significantly increased phage yields at each site (166-fold at Brays Bayou and 520-fold at Clear Creek, Figure 2F and H). Using second stage concentrates, we screened a panel of patient isolates of different genera to compare hits and found more lytic phages at Brays Bayou (7/12 species) than Clear Creek (3/12 species), phenotypically validating our 16S-based mapping strategy (Figure 2G). We employed an identical filtration train at Galveston Bay sampling the marine, saltwater biome. We tracked concentrations with *Vibrio parahaemolyticus* 17,802 and could only identify plaques after second stage concentration (Figure 2F-right panel, J), showing utility in salt water. To test whether phage concentration increased without host-selection bias, we used transmission electron microscopy (TEM). We observed a wide range of phage morphologies with intact tails in concentrates, while the input was mostly clear of particles (Figure 2I and K). This provides further objective evidence of unbiased concentration of intact phages through the ΦHD process.

### GEΦMAPPING of wastewater treatment plants in Houston, Texas

**Figure 3.**
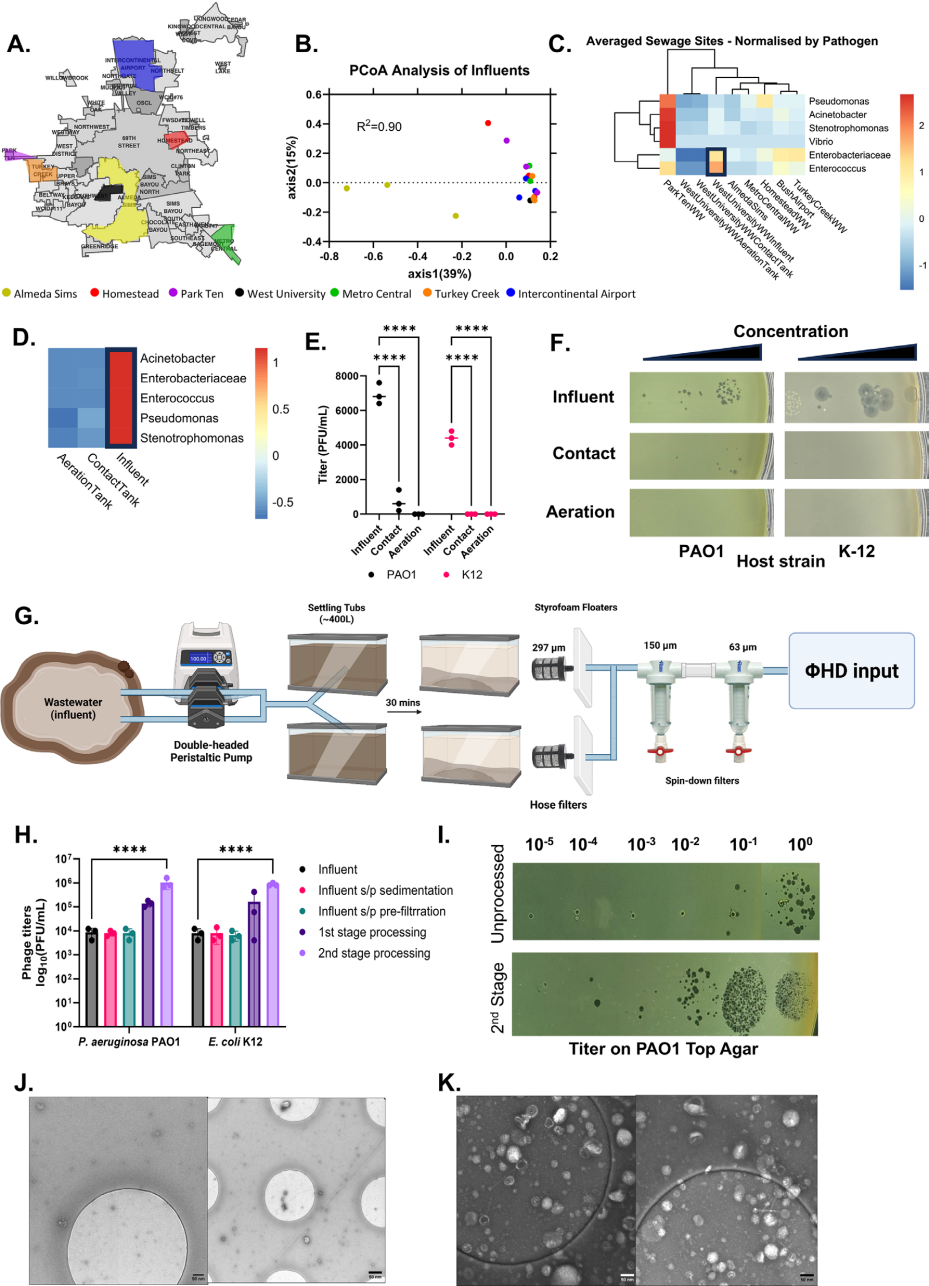
GEΦMAPPING identified *Enterococcus* and *Enterobacteriaceae* was rich at West University WWTP, and performance of ΦHD sampling in wastewater. (**A**) Schematic of geographical areas drained by WWTPs in Houston, TX, USA. (**B**) β-diversity analysis shows variation in OTUs present in WWTPs by PCoA analysis based on Bray–Curtis dissimilarity (AB, colors represent differing WWTPs). (**C**) Heatmap analysis of pathogenic taxa highlights (black box) location of enteric pathogens (z-score normalized by taxa). (**D**) 16 S analysis further localizes pathogenic taxa to wastewater influent (z-score normalized by taxa). (**E**) Quantitation of plaques on index strains confirms 16 S sequencing data. (**F**) Titration of VLPs from different WWTP processing areas on PAO1 (*P. aeruginosa*) and K-12 (*Escherichia coli*) index strains (representative data shown). (**G**) Outline of prefiltration and sedimentation step for wastewater sites. (**H**) Quantification of anti-Pseudomonas plaques and anti-Escherichia plaques from different stages of processing shows phage retention through the system and concentration of the end products. (**I**) Titration of VLPs from different WWTP processing areas on PAO1 (*P. aeruginosa*). (**J**) TEM of unprocessed ΦHD input from wastewater. (**K**) TEM of second-stage concentrated wastewater. Representative images shown (**F, I–K**). Statistical analysis. (**E**) Phage titers across wastewater treatment stages and indicator strains were compared using a two-way ANOVA with Tukey’s multiple comparisons test (*α*=0.05; n=3 technical replicates per condition), revealing significant effects of treatment stage (F(2,12) = 399.3, p<0.0001), indicator strain (F(1,12) = 35.31, p<0.0001), and their interaction (F(2,12) = 16.87, p=0.0003), indicating that the effect of treatment stage differed between indicator strains. Influent yielded significantly higher phage titers than both contact and aeration tanks for PAO1 (p<0.0001 for both) and K-12 (p<0.0001 for both), while no significant difference was detected between contact and aeration tanks for either strain (PAO1: p=0.093; K-12: p>0.9999). (**H**) Phage titers across wastewater processing stages and indicator strains were compared using a two-way ANOVA with Dunnett’s multiple comparisons test (*α*=0.05; n=3 technical replicates per condition). A significant effect of processing stage was detected (F(4,20) = 31.68, p<0.0001), while neither indicator strain (F(1,20) = 0.188, p=0.669) nor the interaction between strain and processing stage (F(4,20) = 0.262, p=0.899) reached significance, indicating that processing stage drove phage yield increases independent of indicator strain. Second-stage processing yielded significantly higher titers relative to unprocessed influent for both PAO1 (p<0.0001) and K-12 (p<0.0001), while sedimentation, pre-filtration, and first-stage processing did not differ significantly from unprocessed influent for either strain (all p>0.6). We display the mean and SD of three replicates for both graphs.

Pathogens belonging to *Enterococcus* and *Enterobacteriaceae,* including *Escherichia* and *Klebsiella,* are known inhabitants of wastewater, so we reasoned wastewater would be prime choice for finding phages against these pathogens (Brumfield et al., 2022; Drulis-Kawa et al., 2011). In a similar fashion to freshwater mapping, we sampled influents of seven different wastewater treatment plants (WWTPs) twice to generate geΦmaps of the pathogenic landscape of wastewater plants. Figure 3A shows the catchment area for each WWTP in Houston. The logarithmic shape of rarefaction curves showed we adequately sampled from each site (Figure 2—figure supplement 1A, bottom). PCoA of β-diversity (Bray–Curtis dissimilarity) demonstrated close clustering of sewage samples despite substantial diversity in both human populations and localization of industrial and commercial facilities. Unexpectedly, influent samples collected from Almeda Sims WWTP showed unusual abundance of *Chloroflexi*, known to be important in biodegradation in wastewater bioreactors (Figure 3B, yellow; Bovio-Winkler et al., 2023).

**Figure 3—figure supplement 1.**
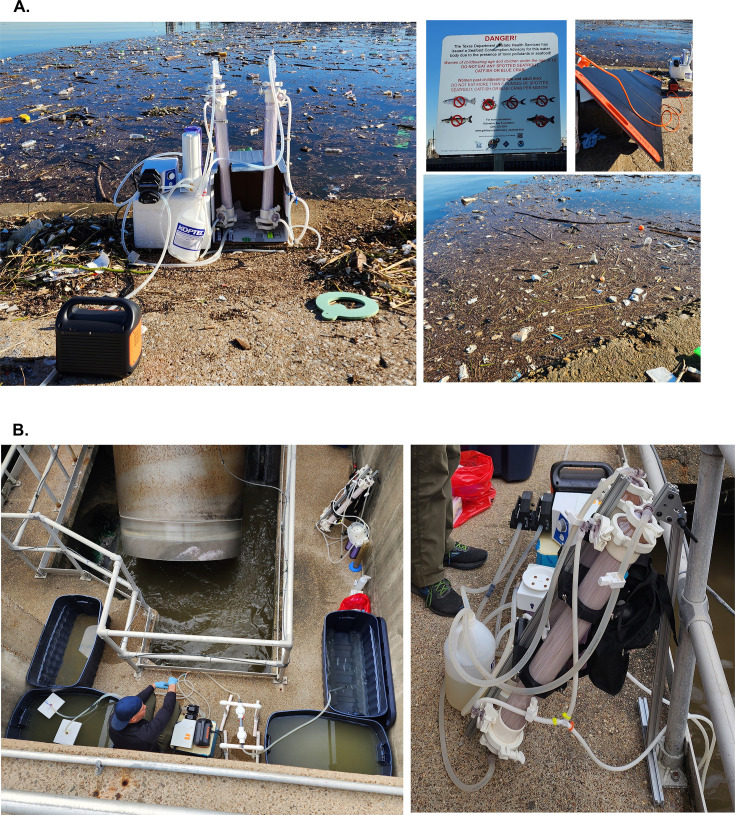
Field photos of ΦHD. (**A**) Buffalo Bayou is accessible at Santa Ana Capture Site. It is relatively close to the Port of Houston where ships enter and exit the Gulf of Mexico. Generally, this location is polluted with trash and dead plant material. There is also a warning sign that cautions women and children to not eat any spotted seatrout, catfish, or blue crab caught in the Bayou due to toxic pollutants present. (**B**) West University WWTP (influent). Raw wastewater enters the plant through large pipes where they are subjected to a large filter (steel bars) to filter hygiene products and trash. In the picture, the influent collects in this container and is propelled upwards with a large Archimedean screw (toward the contact tank).

We normalized by taxa and generated heatmaps to make our wastewater geΦmap (Figure 3C). The Park Ten WWTP influent was rich in *Pseudomonas*, *Acinetobacter,* and *Stenotrophomonas*, but it was being decommissioned during our study. West University WWTP had higher levels of *Enterobacteriaceae* and *Enterococcus*, so we selected it for further investigation. The wastewater treatment process alters the microbiome of wastewater, so we compared the pathogenic taxa throughout the process. We sampled raw wastewater filtered only through large steel bars that remove large debris (influent). Similarly, we sampled wastewater mixed with activated sludge (contact tank) and wastewater exposed to activated sludge with air bubbling (aeration tank; Burton, 2013). α-diversity indices showed that microbial diversity was richest in the contact tank and aeration tank of West University WWTP (Figure 2—figure supplement 1B), but the influent tank had the highest abundance of the selected pathogenic taxa (Figure 3D). We confirmed this 16 S genomic data by comparing titers of phages against indicator strains (PAO1 for *Pseudomonas* and K-12 for *Escherichia coli*) which showed the majority of these phages were found in the influent (Figure 3E–F).

### Wastewater sampling with ΦHD

We used a modified ΦHD method to process 400 L of influent wastewater from West University WWTP (Figure 3G, Figure 3—figure supplement 1). To avoid pump issues caused by gravity, we lifted the wastewater 1–2 m onto an elevated deck into large tubs. We allowed this material to settle for 30 min to sediment large debris and then we pre-filtered the sample using 297 µm hose filters and two spin-down sediment traps (150 and 63 µm screens) to produce a clarified product. This pre-filtered material was then used as the ΦHD input. To ensure these additional filtration steps did not capture significant quantities of phage, we titered the material at each stage against indicator strains (PAO1 and K12) and found negligible losses (Figure 3H). We titered input and second stage concentrates (Figure 3H and I) on *Pseudomonas* and *Escherichia* indicators with final phage yields increasing by 118-fold and 108-fold, respectively. We attempted to look for non-selective concentration of virus-like particles (VLPs) with TEM; however, this was complicated by residual particulate matter. Nonetheless, we were able to appreciate some phages in retentates but not the unconcentrated input material (Figure 3J and K). We also screened the second stage concentrate against the previous panel of clinical and laboratory strains. This yielded phages for 9 of 12 bacterial strains screened, and phage titers were higher than at other sites (darker red; Figure 2G).

### Collective shallow shotgun metagenomic sequencing

**Figure 4.**
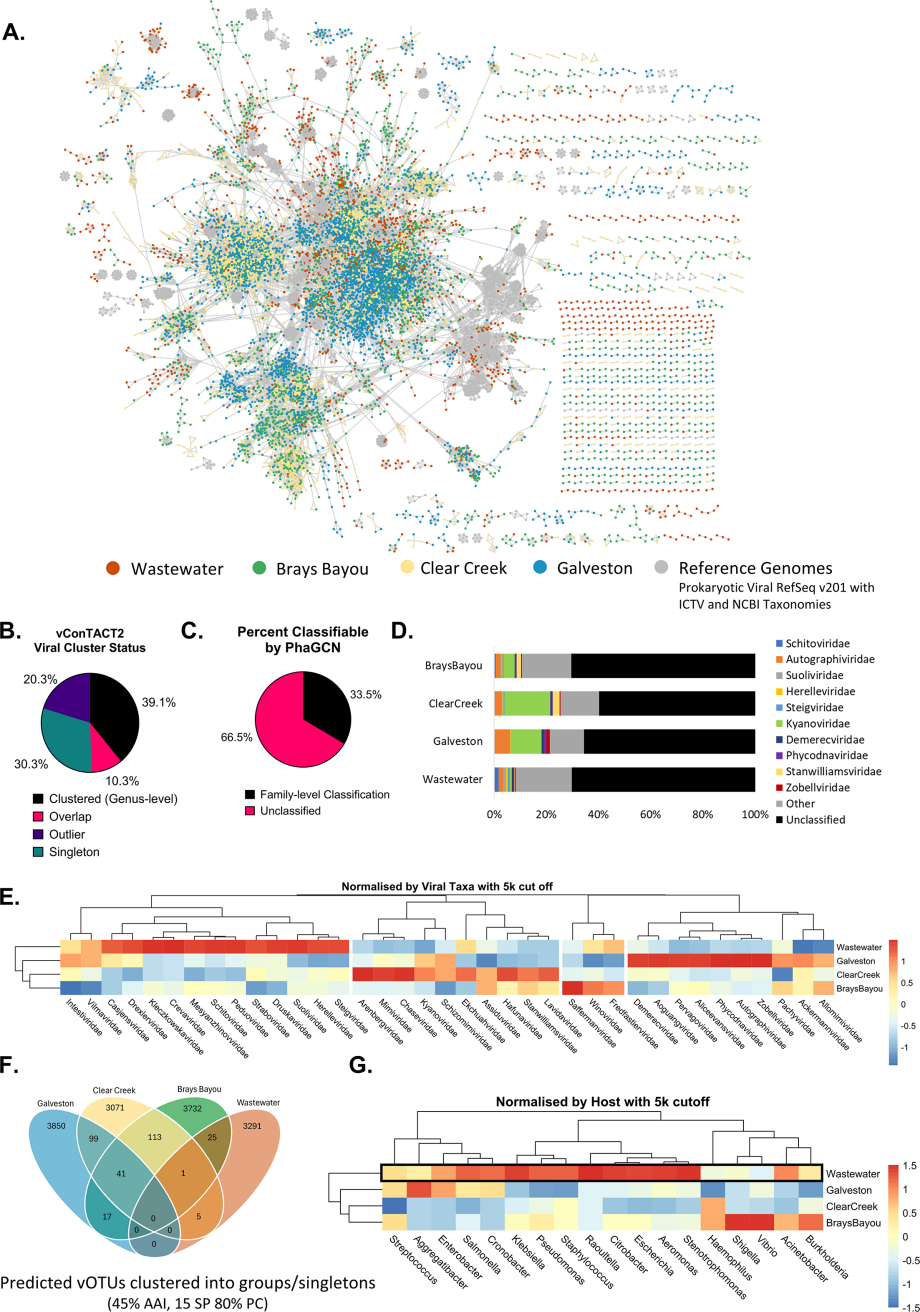
Taxonomic classification of viral contigs from metagenomic datasets reveals distinct viral signatures as well as biased presence of viruses of pathogenic hosts between different locations. (**A**) vConTACT2 gene-sharing network of 16,198 viral operational taxonomic units (vOTUs; ≥5 kb) from West University WWTP, Brays Bayou, Clear Creek, and Galveston. Each dot (node) represents a vOTU, and each line (edge) represents the similarity between each genome. Additionally, 3508 reference genomes from the Prokaryotic Viral RefSeq v201 database are shown in grey. (**B**) Pie-chart of viral cluster status of vOTUs from vConTACT2. (**C**) Pie-chart of classified (family-level) vs unclassified vOTU from PhaGCN. (**D**) Bar graph of family-level classification (PhaGCN counts) of topmost abundant genera across the samples. Remaining genera are grouped together as ‘others’ in grey. Unclassified vOTUs are in black. (**E**) Heatmap with z-score normalized by viral taxa to show distinct viral signatures between sites (pheatmap separated each cluster). (**F**) Venn-diagram of shared vOTUs (cluster mode: amino acid identity [AAI] 45%, protein coverage [PC] 80%) depicts the number of unique vOTUs present at each site and shared between sites. (**G**) Heatmap with z-score normalized by host reveals the relevance of sampling specific sites for phage hunting.

Phage diversity varies significantly across different biomes due to factors such as host availability, ecological niches, and environmental conditions. We sought to compare the viromes of all our ΦHD concentrate (Figure 4). Before that, to test the utility of ΦHD for metagenomic applications, we compared viral metagenomes extracted from an unprocessed sample (5 L) and a ΦHD concentrate (60 mL of 6667 X retentate, corresponding to 400 L of processed sample). The majority of α-diversity indices were similar, suggesting comparable within-sample diversity; however, β-diversity based on Bray–Curtis dissimilarity (0.6062) indicates highly moderate differences in viral community composition. (Supplementary file 6). Heatmap of viral population abundances showed no clear clustering or distinct patterns between the two samples (Figure 4—figure supplement 1A). Nevertheless, we detected substantial increases in unique viral contigs (Supplementary file 7, Figure 4—figure supplement 1B and C). We compared putative *Pseudomonas*-infecting viruses from the input and ΦHD samples, and the ΦHD sample contained both different members from genetically distinct groups and a greater absolute number of unique anti-*Pseudomonas* viruses (Figure 4—figure supplement 1D–F).

**Figure 4—figure supplement 1.**
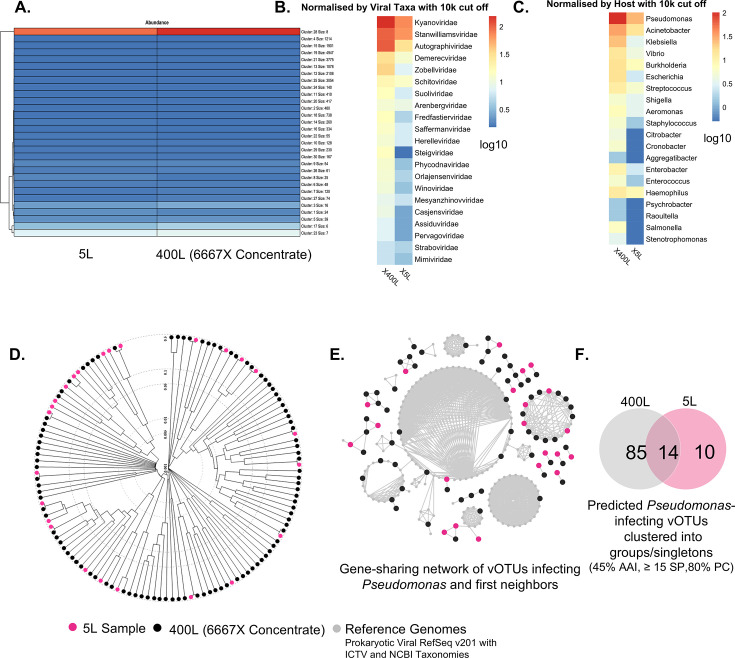
ΦHD concentrate (processed 400 L) has a greater number of viruses present than unprocessed sample (5 L). (**A**) Heatmap of viral population abundances, calculated using z-scores. The 21,918 vOTUs identified from the 400 L and 5 L freshwater samples (Supp. File 8) were used as the reference genomes for MetaPop’s macrodiversity pipeline. (**B**) Heatmap normalized by viral taxa (10 kb cut-off for vOTUs). We classified vOTUs with PhaGCN to the family level and generated heatmaps to visualize the concentration of taxa from a 5 L vs 400 L sample. (**C**) Heatmap normalized by predicted hosts (10 kb cut-off for vOTUs). We predicted hosts of each vOTU and generated heatmaps to visualize the concentration of pathogenic taxa from each sample. (**D**) Phylogenetic tree of all vOTUs predicted to target *Pseudomonas* hosts from 6667 X (100 vOTUs) and 5 L unprocessed sample (25 vOTUs). Greater than half of the vOTUs from the 5 L sample have some relatedness to the vOTUs from the 400 L ΦHD-concentrate. (**E**) vConTACT2 network analysis of clustered vOTUs infecting *Pseudomonas* and first neighbors, including reference genomes. (**F**) Venn-diagram of vOTUs-grouping (cluster mode: AAI 45%, 15 SP, PC 80%) shows shared vOTUs between samples.

**Figure 4—figure supplement 2.**
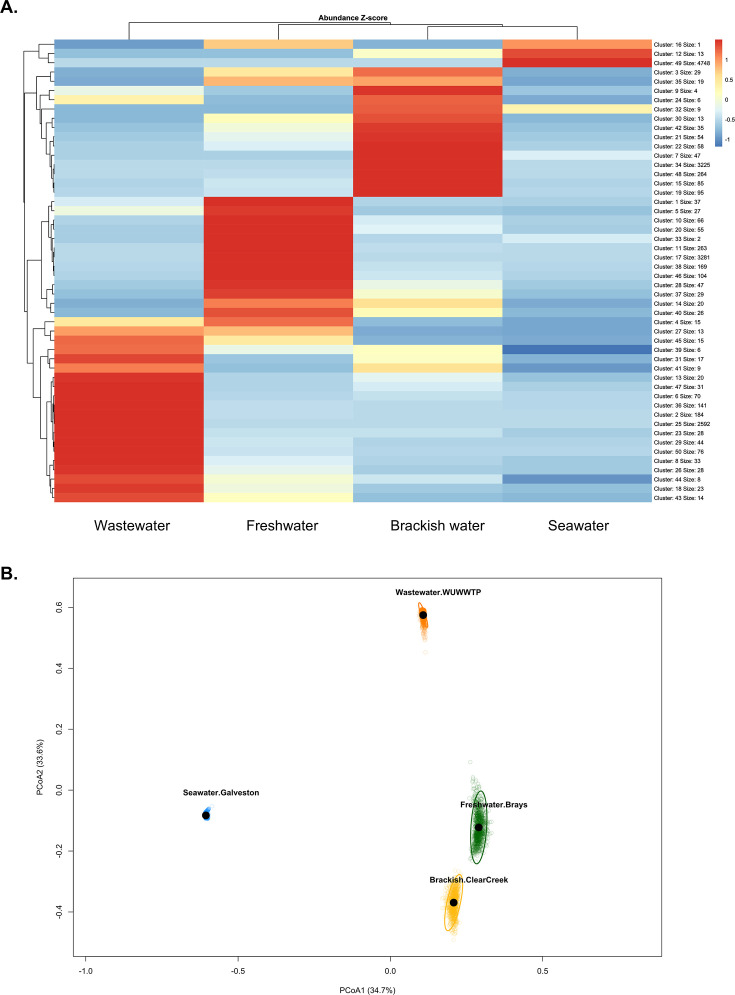
Viral community patterns across biomes. (**A**) Heatmap of z-scored viral population abundances reveals distinct feature-level patterns between biomes. Abundance Files were generated using the MetaPop macrodiversity pipeline with reference genome set as the collection of all vOTUs identified across the four biomes. (**B**) PCoA (based on Bray–Curtis dissimilarity) compares viral communities in seawater (blue), freshwater (green), brackish water (yellow), and wastewater (orange). For PCoA, 2000 features were randomly subsampled and analysis repeated across 1000 bootstrap iterations. Resulting ordinations were aligned to a reference with Procrustes alignment. Mean coordinates and standard deviations were calculated for each sample.

Given the apparent utility of ΦHD in concentrating samples for metagenomics, we looked at the viromes of fresh (Brays), brackish (Clear Creek), waste (West University WWTP), and seawater (Galveston). Based on α-diversity indices, either freshwater or wastewater samples represent the richest sources of viral diversity (Supplementary file 8). Heatmap of viral abundance and β-diversity analysis with PCoA confirm distinct viral community compositions between biomes (Figure 4—figure supplement 2). We clustered our putative, non-redundant viral sequences into vOTUs, or virus operational taxonomic units. We generated a gene-sharing network with vConTACT2 with each node representing a vOTU, colored by source and clustered them with reference genomes from the Prokaryotic Viral RefSeq v201 database (Figure 4A). We counted 16,198 vOTUs ≥5 kb and 5095 vOTUs ≥10 kb.

39.1% of vOTUs clustered with a reference genome at the genus level, while 20.3% were outliers sharing only one to two genes with other genomes. 30.3% were singletons with no genes related to any other genomes in the data set (Figure 4A and B). We also used PhaGCN to classify each virus, but only 33.5% of viruses could be classified to the family-level (Figure 4C). Of the classified genomes, *Kyanoviridae* (green) and *Autographiviridae* (orange) made up the largest identifiable taxa in Clear Creek and Galveston (Figure 4D). This agrees closely with previous findings that *Kyanoviridae*, containing T4-like viruses that infect cyanobacteria, and *Autographiviridae,* T7-like viruses, have been found in deep-sea viromes (Zheng et al., 2025). Due to current limitations in viromics, a large portion of our metagenomic dataset remains unknown. Collectively, between 60.9% (outlier/singles from vConTACT2) and 66.5% (unclassified from PhaGCN) of vOTUs are in this category. A substantial fraction is considered viral dark matter, but we used the remaining identifiable sequences to provide some biological context. This enables us to validate our sampling strategies and compare samples and biomes from another. We used counts from PhaGCN classifications to generate heatmaps normalized by viral taxa to see distinct viral clusters from each biome type. There are four viral signatures present, separating wastewater, seawater, freshwater, and brackish water (Figure 4E). We further clustered viruses from each biome together to identify overlaps and visualized them with Venn diagrams (Figure 4F). We saw that overlaps of viral genomes reflected the physical environment; for example, the brackish water of Clear Creek overlapped between seawater and freshwater – representing its status as an admixture of the two. Next, we used CHERRY for host prediction and made heatmaps normalized by hosts. A large majority of phages with pathogenic hosts were found in the wastewater virome (Figure 4G, black box).

**Figure 5.**
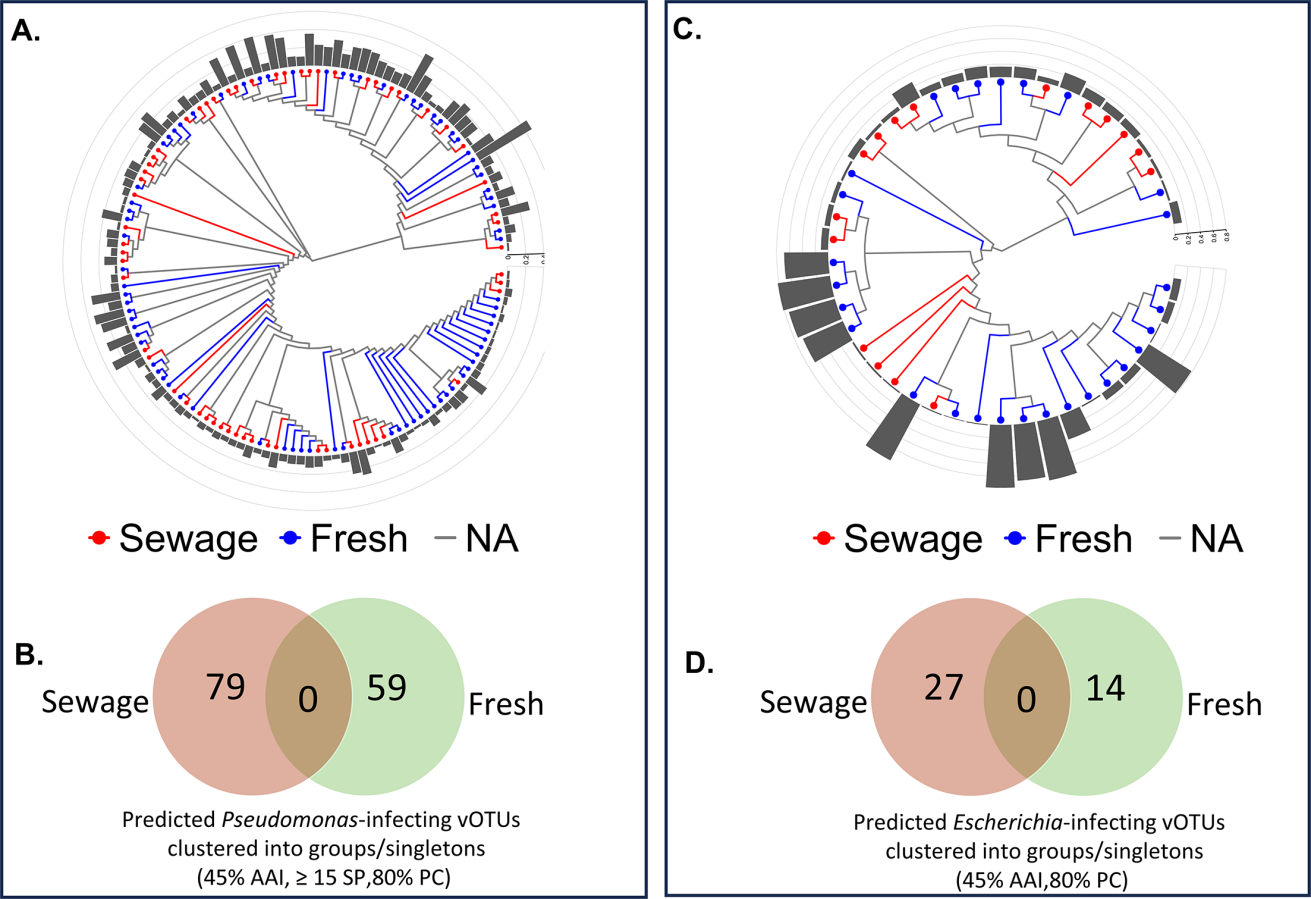
Relevance of multi-site sampling for increased diversity and collection of unique *Pseudomonas and Escherichia* phages. (**A**) Phylogenetic tree of all vOTUs predicted to have *Pseudomonas* host from West University WWTP wastewater and Brays Bayou freshwater metagenomic dataset. Branch-length is non-scaled. (**B**) Venn-diagram of vOTUs-grouping (cluster mode: AAI 45%, 15 shared protein (SP), PC 80%) shows no common vOTUs shared between sites. (**C**) Phylogenetic tree of all vOTUs predicted to have *Escherichia* host from wastewater and Brays Bayou metagenomic dataset. Branch-length is non-scaled. (**D**) Venn-diagram of vOTUs-grouping (cluster mode: AAI 45%, 15 shared protein [SP], PC 80%) shows no common vOTUs shared between sites. Bar graphs (**A, B**) represent the genomic similarities between a contig and its closest neighbor based on genome-wide sequence similarities computed by tBLASTx from VipTree.

To increase phage diversity and minimize the emergence of phage resistance, curating a large, genetically diverse phage library is advantageous. Such a resource allows researchers and clinicians to select phages tailored to their patient needs, including targeting specific phage receptors, maximizing host coverage, designing phage cocktails, or reducing dependence on any single phage (Markwitz et al., 2022). We compared *Pseudomonas*- and *Escherichia*-infecting viruses from freshwater and wastewater. Phylogenetic analysis of *Pseudomonas*-infecting viruses showed significant increases in diversity when combining vOTUs from the two biomes (Figure 5A). Counting and clustering the unique genomes, there were no sequences that were in common between sewage and freshwater (Figure 5B). Analyzing the *Escherichia*-infecting viruses, phylogenetic diversity was greatly increased by including freshwater phages, and the two biomes had no sequences in common (Figure 5C and D). This shows the benefit of creating a phage library using ΦHD from multiple biomes.

### Resistant phage library (RΦ-Library)

**Figure 6.**
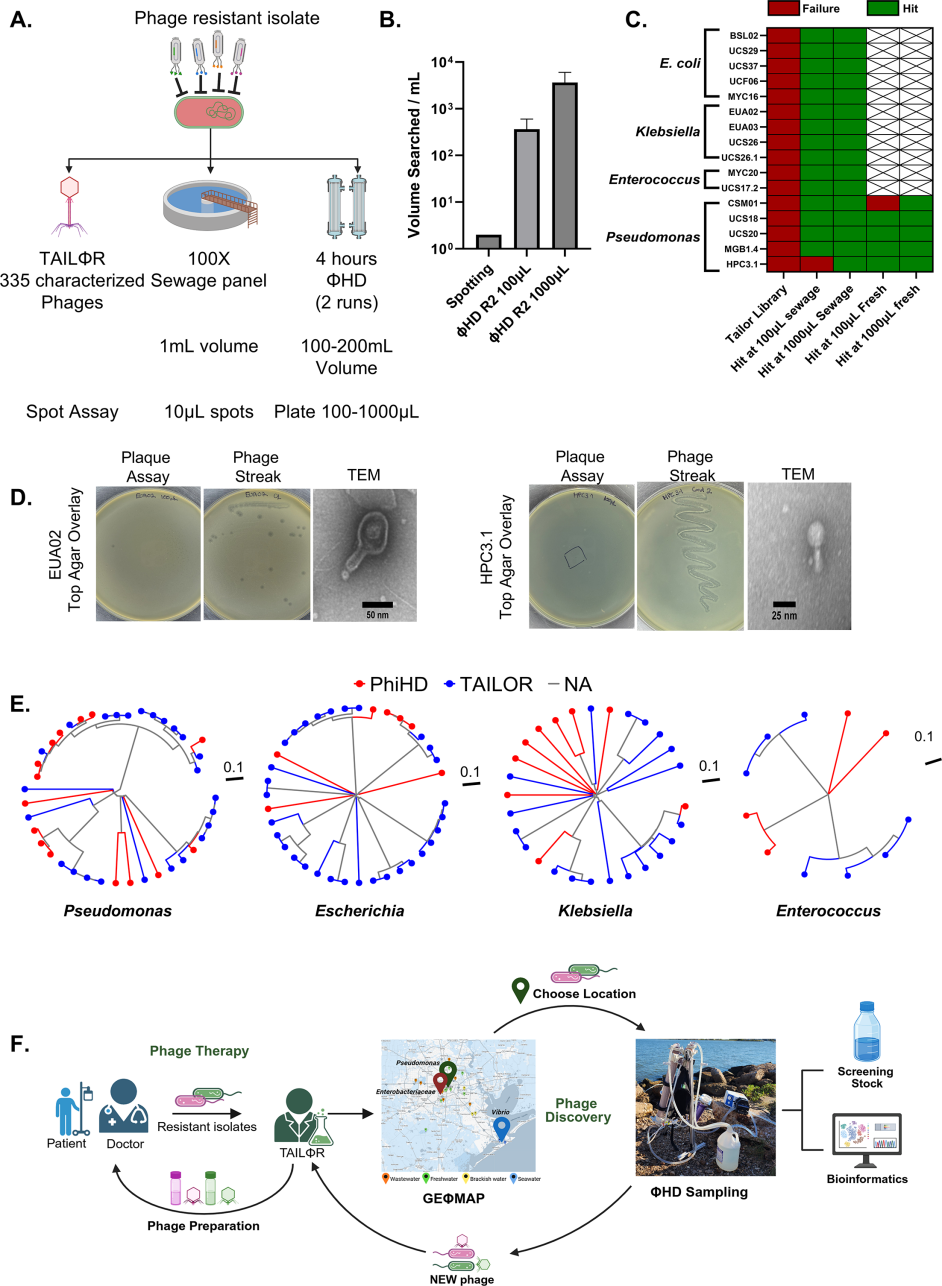
ΦHD increases search volumes and allowed efficient discovery of phages for phage-resistant bacterial pathogens. (**A**) Schematic of ΦHD compared to standard identification and discovery of therapeutic phages. (**B**) Effective volumes that can be searched comparing standard spotting of concentrates with high-concentration, high-volume ΦHD retentates (n=2 retentates with differing final concentrations). We display the mean and SD of two replicates. (**C**) Heatmap showing success/failure of ΦHD compared to the TAILΦR library in finding therapeutic phage candidates. (**D**) Plaque assays (left column) on phage-resistant strains EUA02 (*K. pneumoniae*) and HPC3.1 (*P. aeruginosa*). Individual plaques (middle columns) were streaked and isolated from plaque assays. TEM images (right columns) of select isolated phages infecting EUA02 and HPC3.1. Representative images shown. (**E**) Dendrograms of all isolated phages for *Pseudomonas*, *Escherichia*, *Klebsiella*, *Enterococcus*. Phages from ΦHD are in red, and phages from the TAILΦR library in blue. (**F**) Diagram of the implementation of ΦHD and geΦmapping to the phage discovery pipeline. Created with BioRender.com.

Last-line antibiotics serve as a critical safety net for treating life-threatening infections by multi-drug-resistant bacteria. Their use is restricted to preserve efficacy and slow the development of drug resistance. In a similar manner, the RΦ-library was conceptualized as a last-line phage library to combat extremely phage-resistant organisms (XΦROs). To test our approach of geΦmapping and ΦHD and generate a therapeutic phage library for XΦROs that posed clinical challenges, we used 17 XΦROs from the TAILΦR collection. These failed all conventional phage discovery methods (Figure 6A), including sewage spotting and Appelmans protocol (for host range expansion). As screening ΦHD concentrates allows searching far larger volumes (Figure 6B), we immediately found therapeutic phage candidates for all 17 XΦROs using freshwater (Brays Bayou) and sewage (West University) sources (Figure 6C). We purified (Figure 6D) and characterized 36 new phages by genomic analysis and TEM (Figure 6D and E, Supplementary file 9). This collection of phages is what we termed the RΦ-library. Compared to TAILOR’s sequenced phages, proteomic dendrograms (Figure 6E) showed phages with both subtle and high genetic variation, including new genera exemplified by MYC30C1 and MYC30C2 against *Enterococcus* (Figure 6—figure supplements 1 and 2). Our technique also isolated the jumbo phage UCS29C1 against *E. coli*, which was closely related to the *Klebsiella* jumbo phage Miami, showing that even large phages can survive the process intact (Figure 6—figure supplements 1 and 2; Mora et al., 2021). These successes make GEΦMAPPING and ΦHD sampling excellent additions to TAILΦR’s phage discovery pipeline, allowing for isolation of novel phages with greater precision and efficiency (Figure 6F).

**Figure 6—figure supplement 1.**
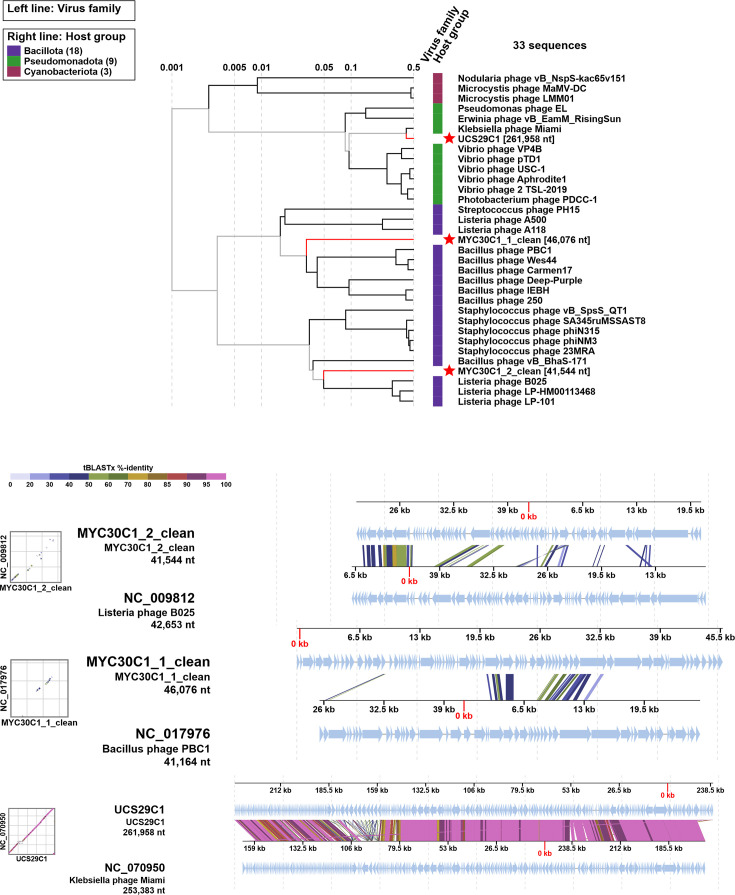
Unique phages isolated from GEΦMAPPING and ΦHD sampling. Proteomic clustering (VIPTree) of UCS29C1, MYC30C1_1, and MYC30C1_2 with their closest neighbor found through BLASTn to look at how distinct their genomes are (*top panel*). Aligned phages with their closest neighbor to depict their percent identity (*bottom panel*).

**Figure 6—figure supplement 2.**
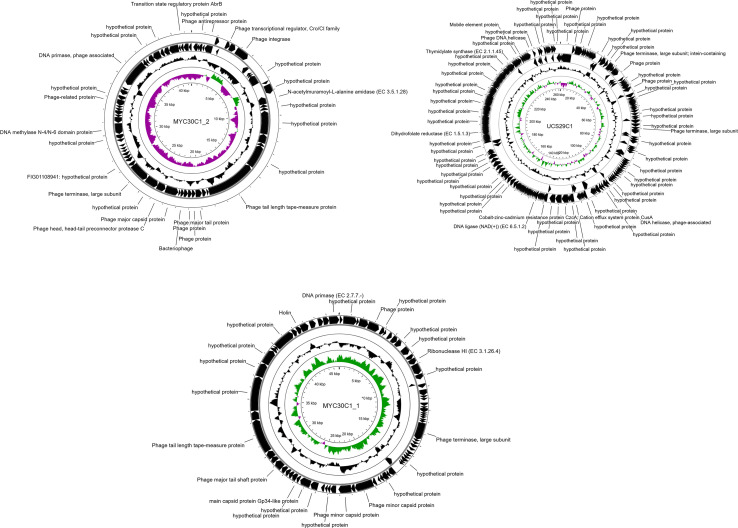
Annotated genomes of unique ΦHD-isolated phages UCS29C1, MYC30C1_1, and MYC30C1_2. Organization of UCS29C1, MYC30C1_1, and MYC30C1_2 genomes. Circular genome map was displayed with CGView. Genomes were annotated using *RAStk*. Coding sequences (CDS, black) are depicted on the two outer rings. *GC content* is shown in black in the middle ring, while *GC skew* is represented on the two innermost rings, with positive skew in green and negative skew in purple.

## Discussion

Our study leverages the billion-year-long evolutionary war between phages and their hosts, focusing on discovering and harnessing natural phages. We report: (i) the design, construction, and successful use of a high-throughput phage capture device (ΦHD); (ii) the use of this device to identify regional sources of both bacteria and phages (geΦmapping); (iii) the use of geΦmapping coupled with ΦHD sampling to identify novel phages that target phage-resistant strains when all other phage matching solutions failed (RΦ-Library); and (iv) the characterization of new metagenomes. Collectively, this work provides a rich resource for therapeutic phages against resistant ESKAPEs (***E****nterococcus faecium, **S**taphylococcus aureus, **K**lebsiella pneumoniae, **A**cinetobacter **b**aumannii, **P**seudomonas aeruginosa, and **E**nterobacter* spp.) and a rich gene library for new biological discovery (Figure 6F).

Wastewater, areas of high human activity, and dense urban development are particularly rich in phages capable of targeting antibiotic-resistant pathogens (Sada and Tessema, 2024; Aghaee et al., 2021; Blanch et al., 2006; Piracha et al., 2014; Chan et al., 2016). This is largely due to the elevated microbial loads in these settings, which naturally support a higher abundance and diversity of phages (Gulino et al., 2020; Olsen et al., 2020a). Human activity imposes evolutionary pressure on bacteria, driving the emergence of antibiotic-resistant strains—and, by extension, their phage counterparts (Pruden et al., 2012). To strategically identify optimal sampling locations, we employed geΦmapping to pinpoint environments with high pathogen loads (Figures 2E and 3C). We identified Brays Bayou as an excellent locale for anti-*Pseudomonas* and other phages, with agreement between genomic mapping and phenotypic phage surveys. We extended our approach to sewage, not only localizing to a single WWTP for phage hunting but to a specific section of that plant (Figure 3A–F).

This targeting enabled the use of ΦHD, which produced phage-rich retentates with phage yields increasing by 100- to 3500-fold each sample in comparison to unprocessed input (Figures 2F and 3H, Figure 2—figure supplement 2G). These retentates were used to target XΦROs in clinical cases resulting in not only the generation of the RΦ-library (Figure 6C) and discovery of new genera of phages (Figure 6—figure supplements 1 and 2) but also treatment in patient cases. Currently, one patient has received two phages that necessitated the ΦHD approach, and six more patient cases are in progress that required ΦHD-derived phages (not published). This illustrates the critical need for phage discovery being addressed by the geΦmapping and ΦHD approach.

Our study is limited by multiple factors. Our current geΦmap is limited to the Gulf Coast area around Houston, TX, USA, and thus is of limited utility to others. However, the overall approach of localizing phage reservoirs is generalizable. With regards to the geomap-guided phage hunt, we only compared one low host-availability control site (Clear Creek) with two high host-availability sites (wastewater and Brays Bayou). More comparisons, especially incorporating even more sites with varied host availability and including additional hosts to screen, would strengthen the claim. Beyond our control, we were limited by weather conditions, as temperature and rainfall can drastically alter both microbial and viral composition. In turn, this limited our sampling time to days with consistent weather (i.e. dry, warm days) for accurate comparisons. In the field, it is technically challenging to remove all phages and bacteria from the hollow fiber units at the end of a sampling run. We employ simple backflushing through the retentate port, which increases phage yield by 25–50%. More phages could be stuck in the hollow fibers. Given the amount of particulate removed later during cleaning, we are certain recovery percentages can be further increased with additional backflushing. While the ΦHD system is portable, the total assembly is large and requires at least two people to carry and operate. We are currently developing a smaller, portable version for single-person use to address this. This would allow for researchers to sample in more remote locations and require less manpower. Finally, our approach is limited to aquatic biomes, but it is well known that the soils of forests and other areas (animal waste, sand, and so on) harbor huge phage biodiversity. We are currently working on ways to address this for both viral ecology and clinical phage discovery.

Collectively, we used a bioprospecting approach to localize therapeutic phages and harvest them en masse with ΦHD. This resulted in large metagenomic data sets informing the viral ecology of the Gulf Coast, critical therapeutic phages for challenging clinical cases, and tools for others to use in phage discovery.

## Methods

### Sample acquisition and preparation for 16S mapping

4 L freshwater and seawater samples from 15 locations were collected in containers cleaned with 10% bleach and copiously pre-rinsed in ddH_2_O. Locations and weather were precisely recorded (Supplementary file 10). Maps were made with EasyMapMaker, 2024. Samples were collected in triplicates with the exception of Brays Bayou @ HEB. Based on 16 S mapping and data not shown here, this location is biologically equivalent to Brays Bayou @ Medical Center due to its short distance and lack of inflow between them. For sampling purposes, we used this location due to its easier access to the bayou and safety reasons. Samples were transported back to the laboratory within hours of gathering samples and stored at 4 °C until further processing the next day. We centrifuged approximately 400 mL of each sample at 10,000 × *g* for 20 min to pellet bacteria and debris. 250 mg was measured out in pre-weighed tubes. In some samples, where there were insufficient pellets (e.g. Buffalo @ SACS, Galveston, West Bay, Sims Bayou, Halls @ Keith Weiss Park), we centrifuged an additional 400 mL of water until 250 mg was met. Sewage samples were obtained from the influent tank of various wastewater treatment plants by plant workers and transported to BCM and handled in a similar fashion. We centrifuged 50 mL wastewater influent at 10,000 × *g* for 20 minutes. 250 mg of the pellet was measured in pre-weighed tubes.

### DNA preparation and 16S processing

DNA was extracted using the DNeasy PowerSoil Kit (Qiagen, Germany) according to the manufacturer’s instructions. Quality was evaluated by A260/280 ratio using a NanoDrop ND-1000 spectrophotometer (Thermo Fisher, USA). Samples with an A260/280 ratio outside of the 1.8–2.0 range were further processed using a Monarch Genomic DNA Purification kit (NEB, USA) using the ‘clean-up’ protocol in the manufacturer’s instructions. Samples were reprocessed from the start if DNA concentrations were insufficient. Samples were sent for sequencing to Novogene. The V4 region of the 16 S gene was amplified using the 515 F (5′-GTGCCAGCMGCCGCGGTAA-3′) and 806 R (5′-GGACTACHVGGGTWTCTAAT-3′) primers (Apprill et al., 2015; Parada et al., 2016), and then sequenced on a Novaseq6000 and 100 K raw reads collected (Novogene, China). We included negative controls with each sequencing batch, consisting of the PowerSoil kit without sample to generate a ‘kitome’ to assess the laboratory and kit background. For these control samples, 16 S rRNA amplification and sequencing was completed despite lack of high-quality DNA inputs. Raw sequences were uploaded to SRA (BioProject #: PRJNA1309115).

### 16S data analysis

Raw reads were processed in mothur (v1.48.0) guided by the MiSeq SOP using SILVA (v132) reference files and seed (Schloss et al., 2009; Kozich et al., 2013; Yilmaz et al., 2014; Quast et al., 2013). Owing to the size, number of samples, and relative lack of computational resources, we used the cluster.split command with taxlevel = 4 to cluster the sequences into OTUs. We generated rarefaction curves using default parameters and calculated alpha-diversity statistics in mothur, subsampling 50,000 random sequences from each group. Beta diversity analysis and PCoA was likewise performed in mothur using Bray–Curtis distance matrices and visualized in Graphpad Prism (v.10.1.2, GraphPad Software, USA). Using R, we performed AMOVA to confirm our β-diversity comparisons. To analyze and visualize the identified taxa in each sample, we generated Krona charts on a Galaxy web server (Ondov et al., 2011; Abueg et al., 2024). We generated heatmaps of the relative amounts of specified taxa by calculating z-scores in R (R Development Core Team, 2010). We calculated z-scores both by site and by taxa, and displayed both using Kolde, 2018.

### ΦHD environmental sampling

A filtration/concentration device, ΦHD, was designed to filter microbes and phages from water. The filtration train starts with a 297 µm stainless steel mesh hose filter (3.6’’ in height and 2’’ in width) attached to silicone peristaltic tubing and goes into a water source (the feed line). The hose filter excludes trash, plant matter, and fish from the system. The tube connects to a Countertop Filtration System equipped sequentially with a 25 µm blown polypropylene cartridge and a 5 µm spun polypropylene cartridge. The filters remove more debris (soil, sand, etc.) and larger microorganisms like bacteria. Tubing then connects to the feed port of a 100 kDa hollow fiber unit (modified polyether sulfone (mPES), 65 cm, KrosFlo Hollow Fiber Filter Module). Anything smaller than 100 kDa (viruses, smaller bacteria, etc.) is filtered by TFF and filtered through the permeate ports. The retentate port ejects filtered water into a (pre-cleaned with 10% bleach and diH_2_0-rinsed) 4 L reservoir. Stop valves are connected to the retentate line and bottom permeate line to control flow rates and fill the jacket of the unit. We use one head of a dual-headed peristaltic pump to propel water through the filtration train. The pump is powered with a travel-size battery station, Jackery Explorer 300, that was charged with solar panels.

Water was filtered and concentrated into a single reservoir, and a second 100 kDa hollow fiber unit was used to further concentrate the water into smaller volumes. The second head of the pump continuously cycled retentates from reservoir to unit. We used a custom-built aluminum stand that allows easy field use of the hollow fiber units. The concentrated product was expected to be 500 mL to 1 L in volume. ΦHD sampling in-field is the first stage of processing (Figure 3—figure supplement 1). Samples were brought back to the lab in 1–2 hr. We centrifuged the samples, generally 1–2 L, at 10,000 × *g* for 20 minutes and filtered the samples with 0.22 µm vacuum-filters (Steritop filters (PES)). Samples were stored at 4 °C until second stage processing in lab (next day). A smaller version of the 100 kDa hollow fiber unit was used to achieve volumes of 50–200 mL per sample (mPES, 20 cm, MidiKros Hollow Fiber Filter Module). Retentate flow rates are controlled by Luer lock stoppers on the retentate line and bottom permeate line. Concentrations of each sample were recorded accordingly after secondary concentration (Supplementary file 5).

### Cleaning-in-place (CIP) protocol of hollow fiber units (TFF units)

After each ΦHD run, we cleaned the TFF units to regenerate them. For 5–10 min, water was flushed through the permeate ports on both ends of the TFF unit. This reverse flow removes the majority of biological build-up in the units. The outer jacket and hollow fibers were then filled with 0.5 M HCl and left overnight to incubate at room temperature. The next day, we rinsed the units with water then soaked outer jacket and hollow fibers in 0.5 M NaOH overnight. Once rinsed with diH_2_O, the units are ready to be used again. For more polluted waters (such as wastewater), we repeated the CIP protocol. We’ve successfully used the units over 10 times for this study (Supplementary file 5).

### ΦHD wastewater sampling

Wastewater has a high burden of flotsam (toilet paper, wet wipes, and feminine hygiene production, etc.) as well as smaller particulates (Figure 3—figure supplement 1B). We pre-filtered wastewater and used the pre-filtered input as the feed to ΦHD. Two silicone peristaltic tubes drew wastewater 1–2 m from the influent tank of West University WWTP and into two large tubs, pre-cleaned with 70% ethanol and Clorox wipes. This helped avoid gravitation issues such as slower flow rates and battery tripping. Wastewater was allowed to settle for 30 min. This allowed larger, denser material to sediment to the bottom. Two tubes with styrofoam floaters and barbed hose filters propelled water through two spin down filters – containing 150 and 63 µm mesh cartridges. The floaters were essential in avoiding the settled debris at the bottom, and the larger filters helped remove more debris. Wastewater was stored in two pre-cleaned tubs; this became the input for ΦHD where we processed the water as described above. For wastewater processing, an additional valve on the feed line is needed to slow down flow rate. Similarly, we centrifuged the first stage concentrate (1–2 L) at 10,000 × *g* for 20 min and filtered the samples with 0.22 µm vacuum filters. Second stage processing lowered the volume to 150–200 mL.

### Bacterial growth and plaque assays

A table of strains used in the study can be found in Supplementary file 11. From a single colony, bacterial cultures were inoculated in Luria Broth (LB, Sigma-Aldrich) and grown overnight at 37 °C with shaking at 180 rpm. For plaque assays, overnight cultures were suspended in 0.75% LB top agar with 10–1000 µL of ΦHD concentrate and poured onto 1.5% LB plates to solidify. Plates were incubated overnight at 37 °C. Plaques are counted next day. Phage titers were visualized with Graphpad Prism (v.10.6.1, GraphPad Software, USA). For phage streaks, a single plaque on agar plates was picked and resuspended in 25 µL of phosphate buffer saline. Bacterial cultures were suspended on 0.75% LB top agar and poured onto LB plates. Before the top agar solidifies, the resuspended plaque was streaked with a pipette. To isolate individual phages from a plaque, the phage was continuously streaked out until plaque morphologies are consistent (at least twice).

### DNA preparation and shotgun shallow metagenomic sequencing

11.5 mL of second stage concentrates from ΦHD (Supplementary file 5) were centrifuged with a SW-41Ti rotor at 37.5 k rpm for 2 hr to pellet VLPs. Supernatant was removed, and the pelleted viruses are used for DNA extraction using the DNeasy PowerSoil kit (Qiagen, Germany) according to manufacturer’s protocols. The extracted DNA was sent for PCR-free library preparation followed by shallow shotgun metagenomic sequencing on the NovaSeq X Plus system platform using the PE150 strategy (Novogene, Beijing). This resulted in >2 G of raw data per sample with Q30>85. Raw sequences were uploaded to SRA (BioProject #: PRJNA1308632).

### Viral metagenomic analysis

Raw data was uploaded into KBASE (Arkin et al., 2018) and examined with FastQC (0.12.1) (Andrews, 2010) then trimmed with Trimmomatic (v0.36) (Bolger et al., 2014) with default settings (sliding window size 4, sliding minimum quality 15, post tail crop length null, head crop length 0, leading minimum quality 3, trailing minimum quality 3, minimum read length 36; Bolger et al., 2014). Post-trimmed reads were assessed with FastQC (0.12.1). Reads were assembled into contigs with metaSPAdes (v3.15.3); minimum contig length was 300≤2000 bp and k-mer sizes were 21, 33, 55, 77, 99, and 127 (Nurk et al., 2017). Contigs were uploaded into CyVerse for further analysis with VIBRANT (Swetnam et al., 2024; Kieft et al., 2020). VIBRANT was used to identify and extract viral contigs from the dataset; default settings were used (length of bp was 1000, number of ORFs per scaffold was 4). Viral contigs from individual samples were clustered into viral operational taxonomic units (vOTUs) using average nucleotide identity (ANI)-based clustering (95% ANI, 85% alignment coverage) on PhaBOX (Shang et al., 2023). We used CheckV (Nayfach et al., 2021) to assess quality and completeness of vOTUs. vOTUs were annotated with PROKKA (v1.14.5) (Seemann, 2014) on KBASE, and gene-sharing networks were made with vConTACT2 (v0.9.19; Bolduc et al., 2017). Settings were set to default, and the reference database used for cluster taxonomy was NCBI Bacterial and Archaeal Viral RefSeq V201. We visualized the gene-networks with Cytoscape (Shannon et al., 2003). vOTUs were further taxonomically classified with PhaGCN (Shang et al., 2021; minimum length ≥5 kb, 75% amino acid identity [AAI], shared protein 15, and 80% protein coverage) and predicted host of each vOTUs with CHERRY (Shang and Sun, 2022; minimum length ≥5 kb, 75% AAI, shared protein 15, and 80% protein coverage, 90% CRISPRs identity). Again, we generated heatmaps of the relative amounts of identified taxa or predicted hosts by calculating z-scores and displayed them using pheatmap in RStudio (R Development Core Team, 2010; Kolde, 2018).

vOTUs with predicted hosts in the genera *Pseudomonas* and *Escherichia* were manually extracted from the datasets of wastewater (West University WWTP) and freshwater (Brays Bayou). We clustered vOTUs with AAI-based clustering mode on PhaBOX (45% AAI, 15 shared protein, and 80% protein coverage). We used VIPTree (Nishimura et al., 2017) phylogenetic analysis then visualized the resulting Newick trees using ggtree (Yu et al., 2017) in RStudio.

### Statistical analysis of metagenomic sequences

Raw data was uploaded into KBASE and examined with FastQC (0.12.1) then trimmed with Trimmomatic (v0.36) with default settings (sliding window size 4, sliding minimum quality 15, post tail crop length null, head crop length 0, leading minimum quality 3, trailing minimum quality 3, minimum read length 36). Post-trimmed reads were assessed with FastQC (0.12.1).

We used MetaPop to analyze viral metagenomic sequence data at the interpopulation (macrodiversity) level (Gregory et al., 2022). We aligned and mapped filtered, trimmed reads of each biome to our reference genome with bowtie2 (v2.5.4+galaxy0). The 16,198 vOTUs (95% ANI, 85% alignment coverage) from wastewater, seawater, brackish, and freshwater were combined and used as our reference genome. Generated BAM output files from bowtie2 and the reference genome were used as input for MetaPop. MetaPop’s macrodiversity analysis includes raw population abundance, normalized population abundance, and α-diversity calculations. The following analyses were conducted in RStudio using tidyverse, vegan, and pheatmap. From normalized population abundance tables, we calculated z-scores and derived heatmaps to display feature-level patterns. We calculated β-diversity using Bray–Curtis dissimilarity, then we performed PCoA with the ordination of the Bray–Curtis matrix. To assess robustness, 2000 features were randomly subsampled and analysis repeated across 1000 bootstrap iterations. Resulting ordinations were aligned to a reference with Procrustes alignment. Mean coordinates and standard deviations were calculated for each sample, and scatter plots were generated.

### Generation of RΦ collection

We obtained clinical isolates from the collection of TAILΦR Labs at Baylor College of Medicine (BCM). We selected isolates for which no phage had been isolated despite exhaustive searches in sewage spotting and testing of the TAILΦR phage library. Study of these clinical isolates was approved by BCM’s Institutional Review Board (IRB). We grew each isolate overnight in LB media, mixed 100 μL of this overnight culture with 3 mL of 0.7% top agar and either 100 μL or 1 mL of environmental or sewage ΦHD concentrate as indicated. Plates were incubated overnight at 37 °C. Next day, plaques that we considered as lytic in appearance (non-cloudy, cleared zones on bacterial lawns) were picked with a pipette tip and streaked in top agar with the same clinical strain prepared in an identical manner. Plaques were streaked three times to ensure purity. We endeavored to pick two to three different plaque morphologies per clinical isolate for each source (environment or sewage). The purified plaques were picked and mixed in 100 μL of phage buffer (10 mM Tris pH 8.0, 100 mM NaCl, 10 mM MgSO_4_). We then made plate lysates by mixing 50 μL of this phage-laden material with 100 μL bacterial overnight of the indicated strain and 3 mL 0.7% top agar and incubating overnight. These were harvested by scraping the top agar from the plates, rinsing the plate surface with 3 mL phage buffer and centrifuging this mixture at 2200 × *g* for 10 minutes and removing residual bacteria with a 0.22 μm PVDF filter (Merck Millipore, Ireland). These phages were titered using standard methods on 0.75% top agar on the indicated strain. Anti-HPC3.1 phages underwent plate lysate production in a laboratory strain PAO1 owing to the amount of mucus produced, and were subsequently titered on the indicated clinical strain. Any other phages found in this study were noted in Supplementary file 12.

### Genetic analysis of the RΦ collection

We employed various methods of DNA preparation for the phages. For samples with adequate titers, we used the EZNA Universal Pathogen Kit (Omega Bio-Tek, USA) according to the manufacturer’s instructions. Phage strains with lower titers or that failed to produce adequate DNA using the EZNA kit were processed by generation of 9 mL of plate lysate (as above) and centrifugation with a Sw-41Ti rotor at 37.5 k rpm for 2 hr to pellet VLPs. This pellet was then processed using a Qiagen PowerSoil Kit using the manufacturer’s instructions. For DNA samples with ratios outside of 1.8–2.0, we further purified them with the NEB Genomic DNA Purification Kit using the manufacturer’s ‘clean-up’ protocol. Whole-genome sequencing using standard protocols was done by BCM’s CMMR core. We processed short reads data using FastQC (v0.12.1; Yehl et al., 2019) to assess data quality, removed low-quality sequences (Q>30 cut-off), and removed the adapters using Trimmomatic (v0.39; Sada and Tessema, 2024) with default settings. We verified the removal of adapters and quality cutoffs with another run of FastQC. These clean reads were then assembled using SPAdes (v3.15.3; DNA source standard, minimum contig length 200≤500; Bankevich et al., 2012) and annotated using RASTtk (v1.073; default setting). We identified candidate viral sequences from these assemblies using (Brettin et al., 2015) VirSorter2 (discarding sequences with <2 hallmark genes, requiring hallmark genes on all sequences, only output high confidence viral sequences, and requiring viral genes to be annotated; Guo et al., 2021). Sequences with high dsDNA phage scores, >four to six hallmark genes, relatively low cellular gene fractions, and coverage suggestive of high copy numbers above the bacterial host were examined manually, and BLASTn (Altschul et al., 1990) was used to determine phage or not. Confirmed phage sequences were then re-annotated with RASTtk (v1.073) to provide final phage genomes for the RΦ library. We compared TAILOR sequences (pre-existing data) with the sequences of purified phages from the RΦ library. We used VIPTree (Nishimura et al., 2017) for a proteomic dendrogram and then visualized the resulting Newick trees using ggtree (Yu et al., 2017) in RStudio. Several Klebsiella phages that were isolated on related host strains were identical, and so we removed these duplicates prior to visualization. All phage genomes were re-annotated with Pharokka v 1.9.0 (Bouras et al., 2023). Coding sequences were predicted with PHANOTATE (McNair et al., 2019). tRNAs were predicted with rRNAscan-SE 2.0 (Chan et al., 2021). tmRNAs were predicted with Aragorn (Laslett and Canback, 2004). CRISPRs were predicted with CRT (Bland et al., 2007). Functional annotations were generated by matching each CDS to the PHROGs (Terzian et al., 2021), VFDB (Chen et al., 2005), and CARD (Alcock et al., 2020) databases using MMseqs2 (Steinegger and Söding, 2017; Larralde and Camargo, 2023; Larralde, 2022) and PyHMMER (Larralde and Zeller, 2023). Phage genomes were rotated with Dnaapler (Bouras et al., 2024). Contigs were matched to their closest hit in the INPHARED (Cook et al., 2021) database using mash (Ondov et al., 2016). GenBank accession numbers for phage genomes are on Supplementary files 9 and 12.

### Electron microscopy and figures

Various environmental, sewage, and purified phage samples were imaged at BCM’s Cryo-EM Advanced Technology Core Facility. Samples were deposited on grids (Quantifoil 2/2 200 Cu +2 nm ThinC; Quantifoil Micro Tools GmbH, Jena, Germany) and negatively stained with 2% uranyl acetate using standard techniques. Images were collected with either a JEOL 1230 electron microscope (JEOL, Japan) at 80 kV equipped with a 4000 by 4000 Gatan Ultrascan charge-coupled device camera (Gatan, Ametek) or a JEOL 2100 (200 kV) electron microscope equipped with a Gatan 4k x 4k CCD. Images were collected at numerous magnifications, and representative examples were selected and viewed in NIH ImageJ.

## Data Availability

Raw shotgun metagenomic sequences were uploaded to NCBI SRA (Sequence Read Archive) under BioProject PRJNA1308632. 16S rRNA amplicon sequences were uploaded to NCBI SRA under BioProject PRJNA1309115. Genome sequences generated in this study have been deposited in the National Center for Biotechnology Information (NCBI) GenBank database, with accession numbers provided in the Supplementary files 9 and 12. We have uploaded our raw data, annotated genomes, generated data/output, analyzed data, and source data to Dataverse (https://doi.org/10.7910/DVN/ZREY8T). The following datasets were generated: DoC SalazarKC ChangJ ClarkJR TerwilligerAL RuchhoeftP NichollsP MaressoAW 2025Geomapping and PhiHD Sampling MetagenomesNCBI BioProjectPRJNA1308632 DoC SalazarKC ChangJ ClarkJR TerwilligerAL RuchhoeftP NichollsP MaressoAW 202516S Geomapping of Houston, TXNCBI BioProjectPRJNA1309115 NichollsP 2026Phage Genomes - Geomapping and PhiHD ProjectHarvard Dataverse10.7910/DVN/ZREY8T

## Additional files

Supplementary file 1. TAILOR distinct case count and number of isolates in their bacterial library.

Supplementary file 2. TAILOR phage library.

Supplementary file 3. Number of isolates with NO phage in TAILOR’s library.

Supplementary file 4. Various viral metagenomic studies with approximate volume processed and reported viral contigs. A total of 16,198 virus operational taxonomic units (vOTUs) were identified from four specific sites in this studies’ metagenomic datasets. 5095 vOTUs were ≥10 kb. Viral contigs were identified from trimmed and cleaned reads with VIBRANT. vOTUs were clustered with standard thresholds of 95% average nucleotide identity over 85% alignment fraction. Additional selected references are shown to depict total volume sampled, viral contigs/viral populations present, and sampling strategy used.

Supplementary file 5. Environmental sampling summary. Water from various sites have been processed with ΦHD and further concentrated in-lab; volume processed and concentration after second stage processing are listed. Concentrates were titered on indicator strain *P. aeruginosa* PA01. DNA was extracted from 11.5 mL of each concentrate and sent for PCR-free shallow shotgun metagenomic sequencing. After metagenomic analysis, viral contigs were identified and clustered into vOTUs based on 5 kb and 10 kb cut-offs. Raw sequences were uploaded to SRA (BioProject #: PRJNA1308632).

Supplementary file 6. α-diversity analysis for metagenomic datasets of unprocessed (5 L sample) and processed ϕHD concentrate (60 mL 6667 X concentrate) from freshwater. We used MetaPop’s macrodiversity pipeline to calculate diversity.

Supplementary file 7. Viral counts of unprocessed vs. processed concentrates. We compared the viromes of unprocessed and ΦHD processed freshwater samples. ΦHD was used to process 400 L of freshwater from Brays Bayou and further concentrated in-lab. Final concentration was 6667 X, and the total volume was used for DNA extraction. 5 L of unprocessed freshwater was concentrated down to 60 mL and used for DNA extraction. DNA was sent for PCR-free shallow shotgun metagenomic sequencing.

Supplementary file 8. α-diversity analysis for metagenomic datasets of brackish, sea, waste, and freshwater. We used MetaPop’s macrodiversity pipeline to calculate diversity.

Supplementary file 9. Characteristics of sequences assembled from members of the R-Phage library. Nearest neighbors determined by total score (BLASTn), coverage, and identity also from BLASTn output. Determination of assemblies representing the same phage by nucleotide alignment (BLASTn), proteomic clustering (VIPTree), plaque morphologies, and known origins of the host. Phages highlighted with the same color indicate they are identical. Submission of whole genome sequences to GenBank in progress.

Supplementary file 10. Samples collected for GEOMAPPING from environmental and wastewater. Samples collected for 16 S sequencing were collected on the days recorded MM/DD/YYYY. Latitude/Longitude were recorded as well as temperature (High °F/ Low °F) and precipitation (inches). Raw sequences were uploaded to SRA (BioProject #: PRJNA1309115).

Supplementary file 11. Strains used in the study. We include short-hand names, full strain names, characteristics, and references for the strains.

Supplementary file 12. Additional phages found from this study. Nearest neighbors determined by total score (BLASTn), coverage, and identity also from BLASTn output. Determination of assemblies representing the same phage by nucleotide alignment (BLASTn), proteomic clustering (VIPTree), plaque morphologies, and known origins of the host. Phages highlighted with the same color indicate they are identical. Submission of whole genome sequences to GenBank in progress

MDAR checklist

Source data 1. Processed and raw outputs used to generate figures, graphs, and diagrams in this article.

## Appendix 1

**Appendix 1—key resources table:**

Reagent type (species) or resource	Designation	Source or reference	Identifiers	Additional information
Strain, strain background (*Acinetobacter baumannii*)	17978	ATCC 17978 Bouvet and Grimont, 1986		Received from TAILФR LABS; bacterial strain originally isolated from fatal meningitis; Used in the multidrug-resistant isolate panel for phage screening - Figure 2G
Strain, strain background (*Achromobacter xylosoxidans*)	UTS1	Gift-TAILOR Labs		Received from TAILФR LABS; bacterial strain originally isolated from cystic fibrosis (CF) lung infection; Used in the multidrug-resistant isolate panel for phage screening - Figure 2G
Strain, strain background (*Enterobacter cloacae*)	SLC1	Gift-TAILOR Labs		Received from TAILФR LABS; bacterial strain originally isolated from; Used in the multidrug-resistant isolate panel for phage screening - Figure 2G
Strain, strain background (*Enterococcus faecalis*)	VRE001	Gift - TAILOR Labs. El Haddad et al., 2022		Received from TAILФR LABS; Vancomycin-resistant isolate; bacterial strain originally isolated from patient stool; Used in the multidrug-resistant isolate panel for phage screening - Figure 2G
Strain, strain background (*Escherichia coli*)	K12	Gift - TAILOR Labs. Blattner et al., 1997		Received from TAILФR LABS; Wild-type, common laboratory strain; Used in the multidrug-resistant isolate panel for phage screening - Figure 2G; also used as our phage indicator strain (Figure 3E, F, H and I)
Strain, strain background (*E. coli*)	JJ2528	Gift - TAILOR Labs. Price et al., 2013		Received from TAILФR LABS; ExPEC ST131 H30-R; Used in the multidrug-resistant isolate panel for phage screening - Figure 2G
Strain, strain background (*Klebsiella aerogenes*)	UCF1	Gift - TAILOR Labs. Terwilliger et al., 2021		Received from TAILФR LABS; bacterial strain originally isolated from hip prosthesis wound; Used in the multidrug-resistant isolate panel for phage screening - Figure 2G
Strain, strain background (*Klebsiella pneumoniae*)	EUA2	Gift - TAILOR Labs		Received from TAILФR LABS; bacterial strain originally isolated from urinary tract infection (UTI); Used in the multidrug-resistant isolate panel for phage screening - Figure 2G
Strain, strain background (*Pseudomonas aeruginosa*)	DSA497	Gift - TAILOR Labs		Received from TAILФR LABS; Ramig LAB; bacterial strain originally isolated from UTI; Used in the multidrug-resistant isolate panel for phage screening - Figure 2G
Strain, strain background (*P. aeruginosa*)	PA01	Gift - TAILOR Labs. Holloway, 1955		Received from TAILФR LABS; Common laboratory strain; bacterial strain originally isolated from wound; Used in the multidrug-resistant isolate panel for phage screening - Figure 2G; also used as our indicator strain (Figure 3E, F and H)
Strain, strain background (*Staphylococcus aureus*)	AZM28	Gift - TAILOR Labs		Received from TAILФR LABS; bacterial strain originally isolated from dog skin swab #13–1 on MSA; Used in the multidrug-resistant isolate panel for phage screening - Figure 2G
Strain, strain background (*Staphylococcus pseudintermedius*)	AZM69	Gift - TAILOR Labs		Received from TAILФR LABS; Coagulase positive; bacterial strain originally isolated from canine ear infection; Used in the multidrug-resistant isolate panel for phage screening - Figure 2G
Strain, strain background (*P. aeruginosa*)	AR0246	CDC and FDA Antibiotic Resistance Isolate Bank; *Pseudomonas aeruginosa* (PSA) Panel		Received from TAILФR LABS; MDR strain; CDC and FDA Antibiotic Resistance Isolate Bank; Used in the multidrug-resistant isolate panel for phage screening - Figure 2G
Strain, strain background (*P. aeruginosa*)	AR0266	CDC and FDA Antibiotic Resistance Isolate Bank; *Pseudomonas aeruginosa* (PSA)		Received from TAILФR LABS MDR strain; CDC & FDA Antibiotic Resistance Isolate Bank; Used in the multidrug-resistant isolate panel for phage screening - Figure 2G
Strain, strain background (*E. coli*)	BSL02	Gift - TAILOR Labs		Received from TAILФR LABS; bacterial strain originally isolated from UTI; Used in the XΦRO screening panel for phage isolation - Figure 6C;
Strain, strain background (*E. coli*)	UCS29	Gift - TAILOR Labs		Received from TAILФR LABS; bacterial strain originally isolated from UTI; Used in the XΦRO screening panel for phage isolation - Figure 6C;
Strain, strain background (*E. coli*)	UCS37	Gift - TAILOR Labs		Received from TAILФR LABS; bacterial strain originally isolated from urine (pyelonephritis, sepsis); Used in the XΦRO screening panel for phage isolation - Figure 6C;
Strain, strain background (*E. coli*)	UCF06	Gift - TAILOR Labs		Received from TAILФR LABS; bacterial strain originally isolated from bone (infected amputation); Used in the XΦRO screening panel for phage isolation - Figure 6C;
*Strain, strain background* *(K. aerogenes*)	MYC16	Gift - TAILOR Labs		Received from TAILФR LABS; bacterial strain originally isolated from shoulder prosthetic joint infection; Used in the XΦRO screening panel for phage isolation - Figure 6C;
Strain, strain background (*K. pneumoniae*)	EUA02	Gift - TAILOR Labs		Received from TAILФR LABS; bacterial strain originally isolated from UTI; Used in the XΦRO screening panel for phage isolation - Figure 6C;
Strain, strain background (*K. pneumoniae*)	EUA03	Gift - TAILOR Labs		Received from TAILФR LABS; bacterial strain originally isolated from UTI; Used in the XΦRO screening panel for phage isolation - Figure 6C;
Strain, strain background (*K. pneumoniae*)	UCS26	Gift - TAILOR Labs		Received from TAILФR LABS; bacterial strain originally isolated from UTI, bacteremia; Used in the XΦRO screening panel for phage isolation - Figure 6C;
Strain, strain background (*K. pneumoniae*)	UCS26.1	Gift - TAILOR Labs		Received from TAILФR LABS; bacterial strain originally isolated from UTI, bacteremia; Used in the XΦRO screening panel for phage isolation - Figure 6C;
Strain, strain background (*S. aureus*)	MYC20	Gift - TAILOR Labs		Received from TAILФR LABS; bacterial strain originally isolated from sputum (bronchiectasis); Used in the XΦRO screening panel for phage isolation - Figure 6C;
Strain, strain background (*E. coli*)	UCS17.2	Gift - TAILOR Labs		Received from TAILФR LABS; bacterial strain originally isolated from UTI; Used in the XΦRO screening panel for phage isolation - Figure 6C;
Strain, strain background (*P. aeruginosa*)	CSM01	Gift - TAILOR Labs		Received from TAILФR LABS; bacterial strain originally isolated from sputum (lung abscess, adjacent sternal osteomyelitis); Used in the XΦRO screening panel for phage isolation - Figure 6C;
Strain, strain background (*P. aeruginosa*)	UCS18	Gift - TAILOR Labs		Received from TAILФR LABS; bacterial strain originally isolated from bile duct (biliary infection, sepsis); used in the XΦRO screening panel for phage isolation - Figure 6C;
Strain, strain background (*P. aeruginosa*)	UCS20	Gift - TAILOR Labs		Received from TAILФR LABS; bacterial strain originally isolated from chest drain (lung, chest wall infection); used in the XΦRO screening panel for phage isolation - Figure 6C;
Strain, strain background (*P. aeruginosa*)	UCS23	Gift - TAILOR Labs		Received from TAILФR LABS; bacterial strain originally isolated from UTI; used in the XΦRO screening panel for phage isolation - Figure 6C;
Strain, strain background (*P. aeruginosa*)	MGB1.4	Gift - TAILOR Labs		Received from TAILФR LABS; bacterial strain originally isolated from sputum (CF lung); used in the XΦRO screening panel for phage isolation - Figure 6C;
Strain, strain background (*P. aeruginosa*)	HPC3.1	Gift - TAILOR Labs		Received from TAILФR LABS; bacterial strain originally isolated from sputum (lung); used in the XΦRO screening panel for phage isolation - Figure 6C;
Strain, strain background (*Vibrio parahaemolyticus*)	17802	ATCC 17802 Fujino et al., 1974		Purchased from ATCC; isolated from Shirasu food poisoning; used to produce Figure 2F and J (right panel); used to isolate *Vibrio* phages
Strain, strain background (*Vibrio vulnificus*)	27562	ATCC 27562 Reichelt et al., 1976		Purchased from ATCC; isolated from human blood; used as part of our panel to screen concentrated seawater for *Vibrio* phages; did not result in phage
Strain, strain background (*Vibrio parahaemolytic phage*)	VP1	This paper		Novel bacteriophage isolated in this study; isolated from seawater concentrate; strain used to isolate was 17802
Strain, strain background (*V. parahaemolytic phage*)	VP2	This paper		Novel bacteriophage isolated in this study; isolated from seawater concentrate; strain used to isolate was 17802
Strain, strain background (*V. parahaemolytic phage*)	VP3	This paper		Novel bacteriophage isolated in this study; isolated from seawater concentrate; strain used to isolate was 17802
Strain, strain background (*V. parahaemolytic phage*)	VP4	This paper		Novel bacteriophage isolated in this study; isolated from seawater concentrate; strain used to isolate was 17802
Strain, strain background (*Escherichia phage*)	*UCS29C1*	This paper		Novel bacteriophage isolated in this study; isolated from *UCS29*; used to produce Figure 6E
Strain, strain background (*E. phage*)	*UCS29C2*	This paper		Novel bacteriophage isolated in this study; isolated from *UCS29*; used to produce Figure 6E
Strain, strain background (*E. phage*)	*UCS37C1*	This paper		Novel bacteriophage isolated in this study; isolated from *UCS37*; used to produce Figure 6E
Strain, strain background (*E. phage*)	*UCS37C2_1*	This paper		Novel bacteriophage isolated in this study; isolated from *UCS37*; used to produce Figure 6E
Strain, strain background (*E. phage*)	*UCS37C2_2*	This paper		Novel bacteriophage isolated in this study; isolated from *UCS37*; used to produce Figure 6E
Strain, strain background (*E. phage*)	*BSL02C1_1*	This paper		Novel bacteriophage isolated in this study; isolated from *BSL02*; used to produce Figure 6E
Strain, strain background (*E. phage*)	*BSL02C1_2*	This paper		Novel bacteriophage isolated in this study; isolated from *BSL02*; used to produce Figure 6E
Strain, strain background (*E. phage*)	*BSL02C2*	This paper		Novel bacteriophage isolated in this study; isolated from *BSL02*; used to produce 6E
Strain, strain background (*Enterococcus phage*)	*MYC30C1_1*	This paper		Novel bacteriophage isolated in this study; isolated from *MYC30*; used to produce Figure 6E
Strain, strain background (*E. phage*)	*MYC30C1_2*	This paper		Novel bacteriophage isolated in this study; isolated from *MYC30*; used to produce Figure 6E
Strain, strain background (*E. phage*)	*UCS17X2C1*	This paper		Novel bacteriophage isolated in this study; isolated from *UCS17*; used to produce Figure 6E
Strain, strain background (*E. phage*)	*UCS17X2C2*	This paper		Novel bacteriophage isolated in this study; isolated from *UCS17*; used to produce Figure 6E
Strain, strain background (*Klebsiella phage*)	*UCS26C1_1*	This paper		Novel bacteriophage isolated in this study; isolated from *UCS26*; used to produce Figure 6E
Strain, strain background (*K. phage*)	*UCS26C1_2*	This paper		Novel bacteriophage isolated in this study; isolated from *UCS26*; used to produce Figure 6E
Strain, strain background (*K. phage*)	*EUA02C1*	This paper		Novel bacteriophage isolated in this study; isolated from *EUA02*; used to produce Figure 6E
Strain, strain background (*K. phage*)	*EUA3C1*	This paper		Novel bacteriophage isolated in this study; isolated from *EUA03*; used to produce Figure 6E
Strain, strain background (*K. phage*)	*MYC16C2*	This paper		Novel bacteriophage isolated in this study; isolated from *MYC16*; used to produce Figure 6E
Strain, strain background (*K. phage*)	UCS26X1C1_1	This paper		Novel bacteriophage isolated in this study; isolated from *UCS26*; used to produce Figure 6E
Strain, strain background (*K. phage*)	UCS26X1C2_2	This paper		Novel bacteriophage isolated in this study; isolated from *UCS26*; used to produce Figure 6E
Strain, strain background (*K. phage*)	UCS26X1C2_1	This paper		Novel bacteriophage isolated in this study; isolated from *UCS26*; used to produce Figure 6E
Strain, strain background (*K. phage*)	UCS26X1C2_2	This paper		Novel bacteriophage isolated in this study; isolated from *UCS26*; used to produce Figure 6E
Strain, strain background (*K. phage*)	UCS26X1C2_3	This paper		Novel bacteriophage isolated in this study; isolated from *UCS26*; used to produce Figure 6E
Strain, strain background (*K. phage*)	UCS26X1C2_4	This paper		Novel bacteriophage isolated in this study; isolated from *UCS26*; used to produce Figure 6E
Strain, strain background (*K. phage*)	UCS26X1C2_5	This paper		Novel bacteriophage isolated in this study; isolated from *UCS26*; used to produce Figure 6E
Strain, strain background (*K. phage*)	UCS26X1C2_6	This paper		Novel bacteriophage isolated in this study; isolated from *UCS26*; used to produce Figure 6E
Strain, strain background (*K. phage*)	UCS26C2_1	This paper		Novel bacteriophage isolated in this study; isolated from *UCS26*; used to produce Figure 6E
Strain, strain background (*K. phage*)	UCS26C2_2	This paper		Novel bacteriophage isolated in this study; isolated from *UCS26*; used to produce Figure 6E
Strain, strain background (*K. phage*)	UCS26C2_3			Novel bacteriophage isolated in this study; isolated from *UCS26*; used to produce Figure 6E
Strain, strain background (*K. phage*)	MYC16C1	This paper		Novel bacteriophage isolated in this study; isolated from *MYC16*; used to produce Figure 6E
Strain, strain background (*Pseudomonas phage*)	CSM01C1	This paper		Novel bacteriophage isolated in this study; isolated from *CSM01*; used to produce Figure 6E
Strain, strain background (*P. phage*)	UCS20C3	This paper		Novel bacteriophage isolated in this study; isolated from *UCS20*; used to produce Figure 6E
Strain, strain background (*P. phage*)	UCS18C3_1	This paper		Novel bacteriophage isolated in this study; isolated from *UCS18*; used to produce Figure 6E
Strain, strain background (*P. phage*)	UCS18C3_2	This paper		Novel bacteriophage isolated in this study; isolated from *UCS18*; used to produce Figure 6E
Strain, strain background (*P. phage*)	UCS20C5	This paper		Novel bacteriophage isolated in this study; isolated from *UCS20*; used to produce Figure 6E
Strain, strain background (*P. phage*)	UCS20C4	This paper		Novel bacteriophage isolated in this study; isolated from *UCS20*; used to produce Figure 6E
Strain, strain background (*P. phage*)	6X1C4	This paper		Novel bacteriophage isolated in this study; isolated from *6.1*; used to produce Figure 6E
Strain, strain background (*P. phage*)	UCS20C1	This paper		Novel bacteriophage isolated in this study; isolated from *UCS20*; used to produce Figure 6E
Strain, strain background (*P. phage*)	MGB1X4C4	This paper		Novel bacteriophage isolated in this study; isolated from *MGB1.4*; used to produce Figure 6E
Strain, strain background (*P. phage*)	MGB1X4C1	This paper		Novel bacteriophage isolated in this study; isolated from *MGB1.4*; used to produce Figure 6E
Strain, strain background (*P. phage*)	UCS18C4	This paper		Novel bacteriophage isolated in this study; isolated from *UCS18*; used to produce Figure 6E
Strain, strain background (*P. phage*)	MGB1X4C3	This paper		Novel bacteriophage isolated in this study; isolated from *MGB1.4*; used to produce Figure 6E
