# Supplementary material for: Pathogen-phage geomapping to overcome resistance
Source: eLife. 2026 Aug 6;15:RP109259. doi: 10.7554/eLife.109259 (PMC13446902; doi:10.7554/eLife.109259)
Supplement: Peer review history

# Editor report: eLife Assessment

**DOI:** 10.7554/eLife.109259.3.sa0

**Authors:** María Mercedes Zambrano

**Affiliations:** CorpoGen Colombia

**Keywords:** Convincing, Important

This **important** study establishes an environmental sampling workflow for the discovery of bacteriophages capable of infecting antibiotic-resistant pathogens. The authors **convincingly** demonstrate the effectiveness of the approach, even with the limited sampling scheme and the current challenges in viral taxonomy. This study will interest researchers working on bacterial infections, environmental microbiology, and phage-based alternatives for addressing antimicrobial resistance.

# Referee report: Reviewer #1 (Public review)

**DOI:** 10.7554/eLife.109259.3.sa1

[Editors' note: this version has been assessed by the Reviewing Editor without an additional round of formal review from the original reviewers. The authors have addressed the comments raised in the previous round of review.]

Summary:

In the manuscript "Pathogen-Phage Geomapping to Overcome Resistance," Do et al. present an impressive demonstration of using geographical sampling and metagenomics to guide sample choice for enrichment in human-associated microbes and the pathogen of interest to increase the chances of success for isolating phages active against highly resistant bacterial strains. The authors document many notable successes (17!) with highly resistant bacterial isolates and share a thoughtfully structured phage discovery effort, potentially opening the door to similar geomapping efforts across the field. While the work is methodologically strong and valuable for the community, there are a few areas where additional clarification and analysis could better align the claims with the data presented.

Strengths:

(1) The manuscript describes a well-executed and transparent example of overcoming a major obstacle in therapeutic virus identification, providing a practical success story that will resonate with researchers in microbiology and medicine.

(2) Many phage researchers have anecdotally experienced a similar phenomenon, that a particular wastewater treatment plant always seems to have the pathogens you need. Quantifying this with metagenomics modernizes and adds evidence to this phenomenon in a way that could help researchers reproduce this success in a methodical way.

(3) The methodology of combining environmental sampling, viral screening, and host-range analysis is clearly articulated and reproducible, offering a valuable blueprint for others in the field.

(4) The data are presented with appropriate analytical rigor, and the results include robust sequencing and metagenomic profiling that deepen understanding of local viral communities.

(5) The 17 successes yielding 35 phages have a lot of phylogenetic novelty beyond what the Tailor labs have typically found with previous methods.

(6) The work highlights a practical and innovative solution to an increasingly important clinical problem, supporting the development of personalized antiviral strategies.

# Referee report: Reviewer #2 (Public review)

**DOI:** 10.7554/eLife.109259.3.sa2

Summary:

The manuscript by Do and colleagues aims to develop a workflow for isolating and identifying bacteriophages with potential applications in phage therapy against antibiotic-resistant pathogens. The workflow integrates geΦmapping as a strategy to identify potential phage sources, ΦHD as a device for phage concentration, and RΦ as a phage library constructed from the initial sampling, resulting in the discovery of 36 new phages. The paper is overall interesting, and the proposed method appears robust and effective.

Strengths:

The methods proposed combined state-of-the-art strategies to solve an ever-increasing problem of antibiotic resistance. The methods are robust, and the controls are appropriate. The integration of environmental sampling, concentration strategies, and downstream genomic characterization is a clear strength and provides a potentially scalable framework for identifying candidate therapeutic phages. The manuscript is clearly written overall, and the results support the main conclusions.

Comments on revised version:

The manuscript has been adequately improved and adjusted according to the comments. There are minor points such as Table S10 is labelled in the top of the page as Table S11. Also, is a little unconventional to cite result figures and tables in the introduction.

For the question 10, regarding why some of the most abundant vOTUs in the 5L sample were not detected in the concentrate. The answer does not satisfy, as it focuses on why very low abundant vOTUs will not be detected, but the question is why some of the most abundant vOTUs were not detected. This does not affect the results or interpretation made.

# Author comment: Author response

**DOI:** 10.7554/eLife.109259.3.sa3

**Authors:** Camilla Do, Keiko Christine Salazar, James D Chang, Justin R Clark, Austen Lee Terwilliger, Paul Ruchhoeft, Paul Nicholls, Anthony W Maresso

**Affiliations:** Baylor College of Medicine Houston United States; Baylor College of Medicine Houston United States; Baylor College of Medicine Houston United States; Baylor College of Medicine Houston United States; Baylor College of Medicine Houston United States; University of Houston Houston United States; Baylor College of Medicine Houston United States; Baylor College of Medicine houston United States

The following is the authors’ response to the original reviews.

**Public Reviews:**

**Reviewer #1 (Public review):**

(1) The central concept of geomapping as a broadly applicable strategy is wonderfully supported by the 17 successes documented in the paper. While this is actually, of course, a strength, the study does not include a comparative analysis across multiple sites with varying sampling outcomes for different bacterial types, which would be necessary to validate this claim more generally.

We thank the reviewer for the point, and it is well taken. We addressed this below, where we give a full discussion.

(2) Some elements, such as beta diversity comparisons and the metagenomics analysis of viral dark matter, would benefit from additional statistical analysis and clearer context.

The reviewer is quite correct as to the importance of bringing statistical analysis to our metagenomic analysis. To that end, we performed statistical analysis on our metagenomic datasets. We performed statistical analysis on our metagenomic datasets. We approached this using MetaPop to analyze viral metagenomic sequence data at the interpopulation (macrodiversity) level. MetaPop's macrodiversity analysis includes raw population abundance, normalized population abundance, and α-diversity calculations. With normalized population abundance tables, we were able to generate heatmaps to view feature-level distinction between samples and biomes. Furthermore, we were able to calculate β-diversity based on Bray-Curtis dissimilarity. PCoA was performed, and to assess robustness, 2,000 features were randomly subsampled and analysis repeated across 1,000 bootstrap iterations. Resulting ordinations were aligned to a reference with Procrustes alignment. Mean coordinates and standard deviations were calculated for each sample, and scatter plots were generated. Supplementary Tables 6 and 8 and Supplementary Figure 4 have been added.

(3) Claims about therapeutic cocktails would be better framed as speculative and/or moved to the discussion section.

We thank the reviewer for their point, and it is well taken. Please see our more detailed response to this earlier in this reply.

(4) The manuscript could be strengthened by elaborating on the scope and composition of the phage and bacterial isolate collections, which are important for interpreting the broader significance of the findings.

We thank the reviewer for their point. We have added further details on the bacterial and phage isolate collections so the readers may draw the proper conclusions.

**Reviewer #2 (Public review):**

Weaknesses: >

While the authors acknowledge several limitations, some aspects require clearer framing or additional clarification. The proposed workflow focuses exclusively on aquatic environments as sources of phages, which may limit the diversity of hosts and phage types recoverable using this approach. Some interpretations, particularly regarding taxonomic classification and sampling saturation, would benefit from more cautious wording given current limitations in viral taxonomy and the observed data.

The reviewer makes an excellent point. To try and address this, we made several edits to the main text of the discussion section to reframe and add clarification to our limitations. We also mention the limitation of our strategy to aquatic environments. Lastly, we addressed the final sentence below.

**Recommendations for the authors:**

**Reviewer #1 (Recommendations for the authors):**

(1) To really demonstrate geomapping success would require more comparisons: choosing a variety of locations with and without high host levels and then analyzing the yes or no outcomes in terms of whether phages were found. This manuscript demonstrates 17 substantial and significant successes, very much worth sharing, but I am not sure it answers the central question posed in the title and abstract. This could potentially be accomplished through analysis of existing data related to the attempts, or it may be important to state this limitation in the discussion.

We thank the reviewer for their insightful comment on the generalizability of our geomapping strategy and their emphasis on adding more comparisons to support the claim. While we did not test the 17 bacterial isolates (XΦROs) across multiple sites with varied host levels, we did incorporate an initial, broad preliminary screening panel designed to assess the presence and diversity of phage against multiple pathogens and laboratory strains across different sampled environments after site comparisons with the geomap (Fig. 2G). In another instance, we did without the guidance of the geomap (Supp. Fig. 2I). In each case, highly polluted waters located in densely populated areas (wastewater, Brays Bayou, and Buffalo Bayou) had higher success rates in phage recovery compared to other less polluted sites (Clear Creek, Galveston seawater, Hamilton Pool Preserve, and Pedernales Falls). This trend is consistent with previous reports [16,51]. The screening step functioned as an initial comparison and allowed us to gauge phage availability across different genera of bacteria prior to a focused geomap-guided phage hunt. In the geomap-guided experiment, we compared a low host-availability control site (Clear Creek) with two high host-availability sites (wastewater and Brays Bayou). More comparisons, especially incorporating even more sites with varied host availability, would strengthen the claim but not be invalid without it.

In an ideal situation, we would perform the exact additional experiments proposed by the reviewer. However, we ran into logistical issues. As it turns out, phage quantities in various sites vary with weather, specifically rainfall (a finding that we intend to touch on in a future manuscript). As such, this variable must be matched. Even with the long summers of Texas, we were unable to perform multiple, serial PhiHD runs at multiple sites with similar weather conditions. As such we rely on the strength of preliminary screening (16S and plating for phage) to provide a clear guide as to where to place PhiHD experiments.

We would still contend that the consistency between preliminary screening results and subsequent successful geomapping-guided discoveries (finding phages against 17 different isolates) gives sufficient evidence that geomapping is an effective strategy for identifying productive sampling sites. However, we acknowledge the excellent point of the reviewer and we have edited the main text to include this in the limitations, so that the reader may make their own evaluation.

(2) Line 44: 24% of infections >> perhaps better to describe as isolates, as many infections contain multiple isolates of different types. More background in terms of the number of infections in general, comprising the 24% on the bacterial side, and also a description of the 350 phages in terms of known hosts, would be very interesting to add.

This is an excellent suggestion that would add valuable background to the introduction and provide context for phage laboratories interested in the volume of cases handled by TAILΦR and the isolates they receive. We changed the main text to include information regarding this suggestion. We also provided a three extra tables to show (1) TAILΦR’s number of distinct case counts with number of bacterial isolates received, (2) TAILΦR’s phage library, and (3) number of isolates with NO phage. Please see Supp. Tables 1-3.

(3) Line 92: To add a statistical test to the beta diversity comparisons beyond visual inspection of the location of the points on an ordination, I suggest adding a PERMANOVA.

The reviewer makes an excellent point, and we agree that visual inspection should be backed up by rigorous statistics where possible. As such, we performed an analysis of molecular variance (AMOVA, done in Mothur) to compare samples derived from brackish, sea, fresh, and sewage. AMOVA showed statistical significance between the samples and confirms our visual inspection of the β-analysis. The text has been altered to reflect AMOVA data.

(4) Line 112 (related to point 2 and Fig 1A): How many isolates in the collection for which 24% did not have a phage, and 35% were Pseudomonas?

Related to changes in point 2, we added a supplementary table to provide insight into the number of bacterial isolates without a phage. In total, 104 (24% of 435 isolates in the TAILOR library) have no phage, and 35 belong to *Pseudomonas aeruginosa*.

(5) Line 141: How is it known that Pseudomonas phage concentration increased by 95x? There could be unknowns / difficult to cultivate or phages without the right host. Consider describing as yields rather than the absolute concentrations.

We appreciate the reviewer’s point that not all Pseudomonas phages are accounted for due to host specificity and potential unknowns. We agree completely that all we have are surrogates. We tracked the concentration of phages that infected our indicator strain *Pseudomonas aeruginosa* PAO1. We selected PAO1 because of its broad susceptibility profile and its role as a permissive host for isolating a wide range of Pseudomonas phages. While we acknowledge that not all Pseudomonas phages will be detected, PAO1 captures a wide breadth of Pseudomonas phages, enabling consistent comparisons between samples. Our concentration changes reflect a within-sample comparison between unprocessed material and the processed material. Aligned with reviewer’s concern, the values should not be taken as an estimate of absolute phage concentration in the samples. Rather, the values are method-dependent estimates of enrichment efficiency for PAO1-infecting phages. In tandem with PAO1-infecting phages, the concentration of other viral-like particles infecting other organisms is most likely increased with each concentration step. We have revised the entire manuscript to mention “phage yield,” rather than associate an increase to a concentration.

(6) Line 224: 39.1% viruses> I believe this refers to vOTUs rather than viruses.

The reviewer is correct; we appreciate the catch! We corrected the text to “vOTUs.”

(7) Lines 224-230: How does this relate to the expected ~70% Dark Matter?

As observed in many viral metagenomic studies, our dataset is also dominated by viral dark matter. Between 60.9% (outlier/singles from vCONTact2) and 66.5% (unclassified from PhaGCN) of vOTUs are in this group. To proceed with caution, we edited the main text to draw attention to the large percentage of viral dark matter in our metagenomic dataset. Although a substantial fraction of vOTUs is unknown, the remaining identifiable sequences provide some biological context, enable validation of sampling strategies and comparative analyses between samples.

(8) Line 241: there are many perspectives on whether phage treatment should involve cocktails. If a phage is immunogenic and leads to antibody production that can neutralize other phages, one phage could ruin the game for others. Consider presenting this as a perspective, rather than a ground truth, and consider moving to discussion

This is a very insightful input on this perspective. Our intent was not to present this as a definitive conclusion, but rather to highlight a broader need for a more diverse phage library. This is not limited to phage cocktail generation. We changed the introductory sentence to this paragraph to encompass a broader need for phage diversity and succinctly lead into the next sentence.

(9) Figure 2b contains an R2 value of 0.7, and 2c has R2=0.76. Where does this come from? Maybe a PERMANOVA? Please describe in legend and/or methods+results.

Thank you for catching this! β-diversity calculations were based on Bray-Curtis dissimilarity. We have adjusted the methods and results to incorporate this information.

(10) The Rphi library is mentioned in several places, would be wonderful to have a bit more description of this collection.

We thank the reviewer for their sharp eye, we definitely wanted to ensure the reader understands the significance of this. We added some descriptor sentences to better highlight and introduce the RΦ-library.

(11) Consider adding a central success to the abstract, the fact that phages were found for 17 recalcitrant strains of various ESKAPE pathogens, yielding 35 phages after standard phage hunting and experimental evolution approaches had failed.

We appreciate the reviewer for their emphasis on highlighting the success of our manuscript and made the appropriate changes. We added altered the last sentence to the abstract, and added another sentence to summarize our success.

**Reviewer #2 (Recommendations for the authors):**

(1) Figures 1C and 1D require a more detailed description.

We thank the reviewer for noticing this. We have altered the figure legend to be more descriptive.

(2) Raw and assembled sequencing data should be submitted to a public repository, and the accession numbers should be provided.

We have uploaded the raw and assembled sequencing data to a public repository, and the accession numbers are provided in Supp. Table 9 and 12. For raw metagenomic shotgun sequences, BioProject accession is PRJNA1308632 (Supp. Table 2).

(3) Line 89: The text states that the rarefaction curves plateaued; however, by definition, a plateau implies that the curve no longer increases. In the presented data, all curves continue to rise at the final sampling point. This does not affect the conclusions but suggests that sampling saturation has not been fully reached.

This is a great observation by our reviewer. We agree with this point as the curves do not reach a complete plateau. We have revised the text to use more accurate language and clarify sampling depth.

(4) Figure 2D: The heatmap normalized by Z-score within the selected taxa may give a biased impression of enrichment of certain taxa in specific environments, when in fact it only indicates enrichment relative to the other pathogenic taxa included in the analysis.

The reviewer raises a great point, and we should have pointed this out directly. To avoid potential misinterpretations, we have revised main text to explicitly state that the heatmap displays relative enrichment to the other pathogenic taxa.

(5) Given the variable taxonomic resolution achieved by 16S rRNA sequencing (genus or family level), it would be important to highlight that some detected taxa include non-pathogenic members. For example, Vibrio is common in seawater, yet only a few species are pathogenic to humans.

We agree with this! We added a sentence to emphasize this point.

(6) Figure 2G: The color scale bar is uniform across all panels; please adjust for accurate comparison.

For more accurate comparisons between different samples and phage concentrations, we added a second color to assist with visualization.

(7) The PCoA figures should specify which distance metric was used.

We want to thank the reviewer for the catch, we should have mentioned that. Our PCoA was calculated based on Bray-Curtis dissimilarity. We have adjusted the main text and methods section to mention it.

(8) Figure 3: The meaning of the colors in panels A and B should be clarified.

We thank the reviewer for their keen eye. We changed the figure and figure legend to clarify. The colors on the map and PCoA represent influents from various wastewater treatment plants around Texas.

(9) The manuscript jumps from Supplementary Figure 2 to Figure 6. In general, the order and referencing of supplementary materials are confusing. Supplementary tables and figures should not be intercalated within the same file.

We thank the reviewer for their patience and apologize for the confusion. This occurred as we had multiple revisions to the manuscript and we did not update the sequence of the figures. To address the reviewer’s comment, we separated the supplementary tables and figures into apart. We also ensured that each main and supplementary figures and table were mentioned sequentially in the main text.

(10) It is unclear why some vOTUs were observed in the 5 L collection but not in the concentrated sample (10/24; Supplementary Figure 3E). One would expect that the most abundant vOTUs in the 5 L sample should also be easily detected in the concentrate.

The reviewer brings up a fantastic point. One would certainly expect that the most abundant vOTUs in the 5L samples would also be detected in the concentrated sample.

We have several suspicions as to why several vOTUS were not detected in our concentrated samples. Because we used shallow shotgun metagenomic sequencing, as compared to deep sequencing, we may have obscured our ability to detect and quantify low-abundance taxa. Consequently, dominant taxa occupying a large portion of sequencing reads may have masked the detection of rarer species/vOTUs. Lower sequencing depth results in fewer total reads per sample and reduced sensitivity for rare, infrequent species/vOTUs to be detected. When their abundance falls below detecting limits, they may appear absent from a data set.

Furthermore, we reached out to Novogene, who we outsourced for library preparation and shotgun metagenomic sequencing. According to Novogene, not all genetic material in a sample is used during their library preparation. The maximum amount of DNA to build a PCR-free metagenomic library at each time is approximately 1.5 µg of DNA. Although we submitted 184.4 µg of DNA (from the 400L-concentrate) and 25.6 µg of DNA (from the 5L-sample), we suspect only a fraction of the material was used for library preparation and subsequently sequenced. This may have limited the representation of low abundance vOTUs.

(11) Line 227: The statement that "but only 33.5% of viruses could be classified to the family-level" requires caution. Since the traditional Siphoviridae, Podoviridae, and Myoviridae families were abolished, many viruses currently lack family-level classification. Therefore, this taxonomic level may not be ideal for assessing novelty, as many viruses closely related to known types remain unassigned.

We appreciate the reviewer for bringing up this topic. We recognize the current limitation in the viral metagenomic landscape. Many viruses lack-family level classification due to ICTV taxonomic restructuring and the lack of reference genomes present in a database. A large fraction of viral sequences constitute “viral dark matter.” From a single metagenomic dataset, viral dark matter ranges from 60-90% of vOTUs. Within our own dataset, it is also dominated by viral dark matter. Between 60.9% (outlier/singles from vCONTact2) and 66.5% (unclassified from PhaGCN) of vOTUs are in this group. Although a substantial fraction of vOTUs is unknown, the remaining identifiable sequences provide some biological context, enable validation of sampling strategies and comparative analyses between samples. To complement vCONTact2 results, we utilized PhaGCN to classify each vOTU as a means to compare taxa derived from each sampled biome from one another and not to assess novelty of the metagenomic dataset. We aimed to provide measurable and interpretable context to our metagenomes. However, due to the substantial variability and uncertainty in the field and our dataset, we revised the text to highlight the large fraction of unclassified sequences and their implications.

(12) Supplementary Figure 5: The legend does not clearly explain the two inner rings. One may correspond to GC skew, but this should be explicitly stated.

Well spotted! We have made the appropriate corrections.

(13) Line 325: The reference to "50 mL samples" is unclear-please specify which samples this refers to.
